# Internal hydrodynamics within the skeleton of *Acropora pulchra* coral

**DOI:** 10.1016/j.isci.2025.111742

**Published:** 2025-01-04

**Authors:** Yanmei Tian, Pei Zhang, Hui Huang, Liang Lei, Sergio Andres Galindo Torres, Ling Li

**Affiliations:** 1 College of Environmental and Resource Sciences, Zhejiang University, Hangzhou 310058, China; 2 School of Engineering, Westlake University, Hangzhou 310024, China; 3 Key Laboratory of Coastal Environment and Resources of Zhejiang Province, Westlake University Hangzhou 310024, China; 4 Key Laboratory of Tropical Marine Bio-resources and Ecology, Guangdong Provincial Key Laboratory of Applied Marine Biology, South China Sea Institute of Oceanology, Chinese Academy of Science, Guangzhou 510301, China; 5 Tropical Marine Biological Research Station in Hainan, Chinese Academy of Science, Sanya 572000, China

**Keywords:** Earth sciences, Environmental science, Ecology, Mathematics of computing

## Abstract

Many marine life forms, like *Acropora* coral, develop abiotic components to host and support the growth of living organisms. Using numerical models based on real coral samples reconstructed from micro-computed tomography (CT) scan images, we simulated internal flows inside the skeletons of *Acropora pulchra* coral under the influence of ambient ocean currents. The results showed that the coral’s skeletal structure, with specially connected pore space, leads to the flow and material transport within and through the skeleton to assist the coral growth and stability. However, under intensified ocean acidification, the skeletal internal flow may induce the dissolution of aragonite inside the skeleton and weaken the whole coral structure.

## Highlights

• Consistent flow patterns are observed in *Acropora* coral CT scans-based simulations
• Implications of these patterns for coral growth are discussed in detail
• A prediction of coral skeleton dissolution under ocean acidification is presented

## Graphical abstract

**Figure:**
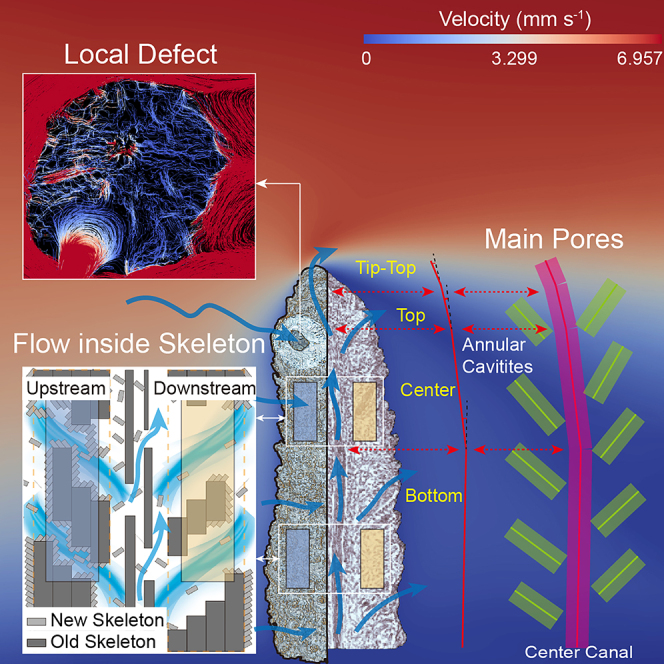

## Introduction

*Acropora* coral is known as one of the most abundant and widespread stony reef-builders in the ocean, with skeletons composed of compact and porous aragonite of excellent mechanical properties. The corallite, distributed across the coral skeleton, serves as the “home” that coral polyps build and cling to. Additionally, coral colonies also serve as the “home” for many other species, such as clownfish and butterflyfish, which provide important habitats in the coral ecosystem.^1^^,^^2^ Increasingly this marine “tropical forest” has become vulnerable to the impacts of global climate change and ocean acidification.^3^ But corals are resilient, even when storms reduce them to rubble, they still possess a significant potential for regeneration. Successful attachment of polyps can initiate recovery approximately 7 months after a storm, although branches may take more than four years to regain their pre-storm growth rates.^4^^,^^5^^,^^6^ The growth of coral is closely related to the flow conditions of seawater at various scales,^7^^,^^8^^,^^9^^,^^10^^,^^11^ and understanding this dependence is important for predicting how coral reefs respond to these stressors.

On the *Acropora* coral surface, polyps attached to the skeleton wave tentacles and beat epidermal cilia to stir the seawater in the diffusion boundary layer (DBL) and generate vortical flows that enhance mass transport for feeding and other active coral physiological processes. At the same time, the delicate structure of their calcified skeleton may be involved in guiding the ambient flow that drives material transport to the DBL.^12^ Across the coral reef, complex canals through coral branches and colonies connect all the polyps, and regulate the flow inside the reef and skeleton, respectively, to sustain and optimize the biological processes.^13^^,^^14^ Here, we suggest that the main structural features of the *Acropora pulchra* coral skeleton are key features affecting the partial flow exchange between the coral internal and the surrounding marine environment. The intricate pore space within the skeleton of a coral branch forms distinct central axial channels and radial corallites,^15^ which may allow orderly and efficient flow distribution inside and outside the coral skeleton under the influence of pressure gradients generated by interactions between the ambient flow and coral branch.^13^^,^^16^^,^^17^ Despite numerous researches on coral tissues and skeletal growth, and significant studies examining the influence of local hydrodynamics on colony growth and biology, a thorough investigation into the internal flow within coral skeleton structures has not yet been conducted. Indeed, coral morphologies are diverse, including encrusting, hemispherical, tabular, corymbose, and branching forms.^18^^,^^19^ All of these morphologies face various environmental challenges, necessitating the development of internal skeletal flows. In this study, we focus on the fastest-growing *Acropora* species, with some of the corals characterized by a branching form morphology and most affected by environmental changes.

The highly complicated pore structure, incredible small pore sizes, and coral tissues covering the surface of the living skeleton make it difficult to observe and measure the flow details inside the coral skeleton even with an experimental high-precision set-up under controlled conditions. Therefore, we used the lattice Boltzmann method (LBM)^20^^,^^21^, a powerful computational fluid dynamics tool, to directly simulate flows around and within a submerged coral branch. The samples were taken from a real skeleton structure of dead *A. pulchra* coral reconstructed by micro-computed tomography (CT) scan. Thus we were able to analyze the potential response mechanism of internal flow’s hydrodynamic characteristics affected by small-scale structural features inside and outside the skeleton. This approach has been applied previously to examine the adaptation mechanism of the skeleton structure of deep-sea sponges living in the abyss.^22^^,^^23^^,^^24^ Based on high-resolution hydrodynamic simulations, we aimed to unravel the links between the complex geometric pore structure^16^^,^^25^ and internal flow characteristics within the *A*. *pulchra* coral skeleton. Our simulations showed that (1) asymmetrical flow caused by inward-extending calyx occurs in interconnected “radial annular corallites”; (2) longitudinal flow (vertical, upward) and transverse (horizontal) flow dominate the “central axial channel” in the top and bottom sections, respectively; and (3) both the emerging vortex and increasing vorticity on the skeleton surface are generated by the outward-extending calyxes. These characteristics, of passive flow (simulated without consideration of living organisms, including coral tissues attached on the skeleton surface), allow for optimal material transport to support polyps growing in different branch sections. Therefore, we suspect that the internal flow guided by the feature of each coral structural subdivision may correspond to a skeleton-flow acclimation mechanism.

Here, we hypothesize the following: (1) the presence of coral biological tissue significantly regulates this exchange of internal and external water flow and material transport over a short period; (2) under the influence of unidirectional water flow, the presence of these biological tissues may not alter the tendency for a pressure gradient to exist within and drive the internal water flow; and (3) in the long term, the passive water flow generated by the pressure gradient within the skeleton will have some impact on the growth of coral polyps. Based on pure coral skeletons, our simulations revealed the exchange characteristics of internal and environmental currents unaffected by regulation of living tissues and organisms attached to the skeleton. Such regulation would restrict the inflow and significantly diminish the internal flow. However, under low pH, our observations, based on passive water movement within dead coral skeletons, may not only help to reveal the coral growth mechanism through the adaptation of the skeleton structure and internal flow to the ambient marine environment, but also to further the understanding of active fluid transfer around the living coral population as a whole, including both polyps and skeletons. Although the skeletal linear extension (upward growth) and densification (lateral thickening) primarily depend on active flow process of the coral polyps in a short period,^25^^,^^26^^,^^27^ there remains a possibility as we hypothesized that the minor internal flow changes, affected by the skeleton structure, may act in combination with coral tissues to influence polyp expansion and coral growth over the long time. Finally, we developed a simple dissolution model to demonstrate that under intensified ocean acidification, internal flow may induce the dissolution of the aragonite within the dead skeleton.^28^ This is particularly relevant for the flow within the skeleton, as it may act as a potential transporter for bioeroders and microorganisms, thereby weakening the entire reef structure. In contrast, living corals exhibit a phenomenon where the pH gradient of aragonite within their tissues increases, thereby protecting the calcification process. Calcium ions dissolved due to acidification can rapidly re-enter the calcified layer through previously overlooked internal pore channels, maintaining a high concentration of calcium ions.

## Results

### *Acropora* coral skeleton structure and general flow features

**Figure 1:**
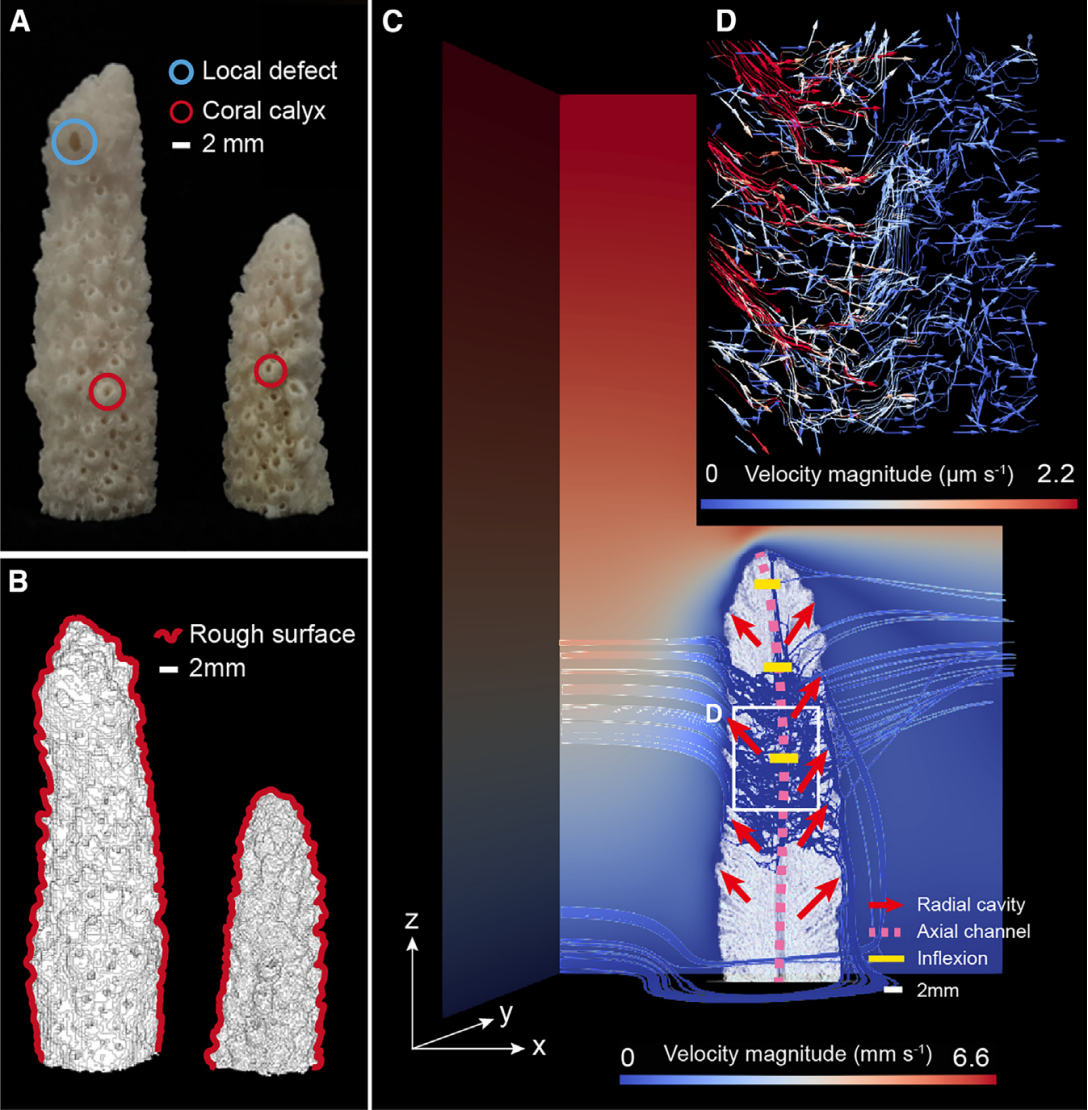
Schematic diagram of the *A*. *pulchra* coral skeleton structure and the associated ambient and internal flow (A) Photo of 2 natural coral branches cut from the *Acropora* coral colony; on the left-hand side is *Sample.1* (S1) with an apparent local defect (blue circle) caused by animal attack and on the right-hand side is *Sample.2* (S2). Red circle indicates calyxes (radial corallites) for coral polyps living. Scale bar: 2 mm. (B) Micro-CT scan images of S1 and S2 with segmentation given by the deep learning modules of ilastik.^87^ CT images cover almost 95% upper parts of the real coral samples. Red curve is the structure contour showing rough coral skeleton surfaces. Scale bar: 2 mm. (C) Simulation domain of S1 with Re = 115, showing the cross-section along x axis through the centreline and the input flow conditions (x = 0). Simulated 3D ambient flow and internal flow through the coral skeleton are also displayed. The streamlines show the flow paths with the color indicating the velocity magnitude. Red arrows are coral interconnected radial annual corallites, pink dash line locates the axial channel, and yellow lines show bending points along the axial channel. Scale bar: 2 mm. (D) Velocity magnitude and direction of the skeletal internal flow for the focused area are selected from (C). The velocity magnitude shows the asymmetry of the flow. The high velocity in the upstream region contrasts with the lower velocity in the downstream, mainly attributed to the flow across the central channel, offering an additional potential exit for the flow.

Corals are vulnerable to storms and hurricanes, which can break them into rubble of varying sizes or even kill them. However, there is evidence that under low flow rates^4^^,^^5^^,^^29^ dead coral branches could reform into living colonies again. Understanding the flow characteristics of dead coral structures may unveil the mechanism under which this rebirth occurs. Through micro-CT scan of dead natural *A*. *pulchra* coral branches (Figure 1A) combined with image reconstruction, and phase segmentation (Figure 1B), and existing structural classification definitions,^15^ we identified the key structural features of the *A*. *pulchra* coral pure skeleton (Figure S1) as follows: (1) rough surface with bumps, (2) radial annular interconnected cavities (radial corallites), (3) central longitudinal axial channel, (4) large and unique corallites at the top tip (axial corallites), and (5) local defects. The two skeleton samples we selected exhibit the basic features (1–4) of *Acropora* coral, with sample 1 notably displaying feature (5). While individual differences among corals should not be overlooked, our analysis focuses solely on the common characteristics. These common features are widely found in coral samples analyzed in previous studies and hence our findings may apply to other corals samples. The second and third features display a skeletal pore space resembling a “tree” structure, reminiscent of a coral colony with a similar branching pattern.

The high-precision scanning pore structure with complex geometric morphologies was used directly to develop models to simulate the ambient flow of seawater with low velocities around the submerged coral branch placed vertically (in its natural orientation) as well as flow within the skeleton driven by pressure gradients due to ambient flow and skeleton interactions space needed. The simulations covered a wide range of realistic ambient flow conditions, with flow rates corresponding to the surface (1 m depth) current velocities in Sanya Bay and velocity range on the surface of coral tissue.^12^^,^^30^ Furthermore, measurements of velocity profiles in similar scenarios have shown that surface velocities on the order of 100 cm/s correspond to velocities at the coral level of 1–10 cm/s.^31^^,^^32^ This is further elucidated by the work of Lowe et al.^32^ where the drag coefficient increases from values of 0.01 above the coral to values of 1 at the coral colony level. This increase in the drag coefficient produces similar decreases in the fluid velocity by a factor of 100. Velocities were characterized by Reynolds number, ranging from 11 to 105, 204, 275, 354, and 500 (Reynolds number is given by Re = |u_inlet_|W/ν with u_inlet_ being the maximum velocity of the inlet flux, simulating the ambient flow, W is the coral width and ν the fluid viscosity; see the STAR Methods section and Table S2 for further details), and revealed flows around and inside the coral skeleton as shown in Figures 1C and 1D.

Based on the average kinetic energy of the entire simulation domain (Figure S5), the velocity components and pressure (Figure S6) at the four locations as shown in Figure S3 together with pressure, velocity, and vorticity changes in the domain (Video S1), we tracked the whole simulation process for the case with Re = 105 (maximum input velocity of ambient flow = 0.85 cm/s) and confirmed the simulation reached a quasi-steady state at the end. Compared with points A and B outside the skeleton, the flow velocity of point D inside the skeleton is substantially smaller, with the magnitude only 0.16‰–1.6‰ of those at the former two points. Meanwhile, flows at the four locations were examined over the simulation duration of 274–285 s (period of quasi-steady state checked and confirmed) for all the cases with different Re values (Figures S7-1 and S7-2). As the Reynolds number increased, the flow velocity oscillated with amplitude increased steadily at locations outside the skeleton while the internal flow speed fluctuated more chaotically during this period, but both became steady toward the middle or late stage of the simulations.

Video S1. The whole simulation process of ambient pressure, ambient velocity and ambient vorticity in S1 at Re = 105

We visualized the ambient flow velocity (Video S2), ambient fluid vorticity (Video S3), internal flow velocity in the skeleton (Video S4), and internal fluid vorticity (Video S5) for all cases of *Sample.1* (S1) and *Sample.1R* (S1R). The skeleton was placed in different orientations horizontally for cases S1 and S1R, with one being a half rotation of the other around the z axis; and the contact surface of flow for S1 was slightly at an acute angle with respect to the x axis while that for S1R was at an obtuse angle.^33^ Thus, according to “extended anisotropy” linked to the asymmetry of the solid morphology and under the same ambient flow condition, turbulence first appeared around the skeleton in case S1 with Re = 354, reflecting the inertial effects on the microscopic velocity structure.^33^ Video S3 shows that the vorticity at the top and bumps of the skeleton is significantly higher than that at other locations, which is closely related to the surface roughness. The velocity and vorticity in Videos S4 and S5 showed the asymmetry of the flow inside the skeleton and higher flow velocity at the top than those at the bottom. Moreover, the internal flow inside the skeleton was affected by the turbulence.

Video S2. The simulation process (274–285 s) of ambient velocity in S1, S1R at Re = 11, 105, 204, 275, 354, 500

Video S3. The simulation process (274–285 s) of ambient vorticity in S1, S1R at Re = 11, 105, 204, 275, 354, 500

Video S4. The simulation process (274–285 s) of inner velocity in S1, S1R at Re = 11, 105, 204, 275, 354, 500

Video S5. The simulation process (274–285 s) of inner vorticity in S1, S1R at Re = 11, 105, 204, 275, 354, 500

### Asymmetric radial flow inside the skeleton

**Figure 2:**
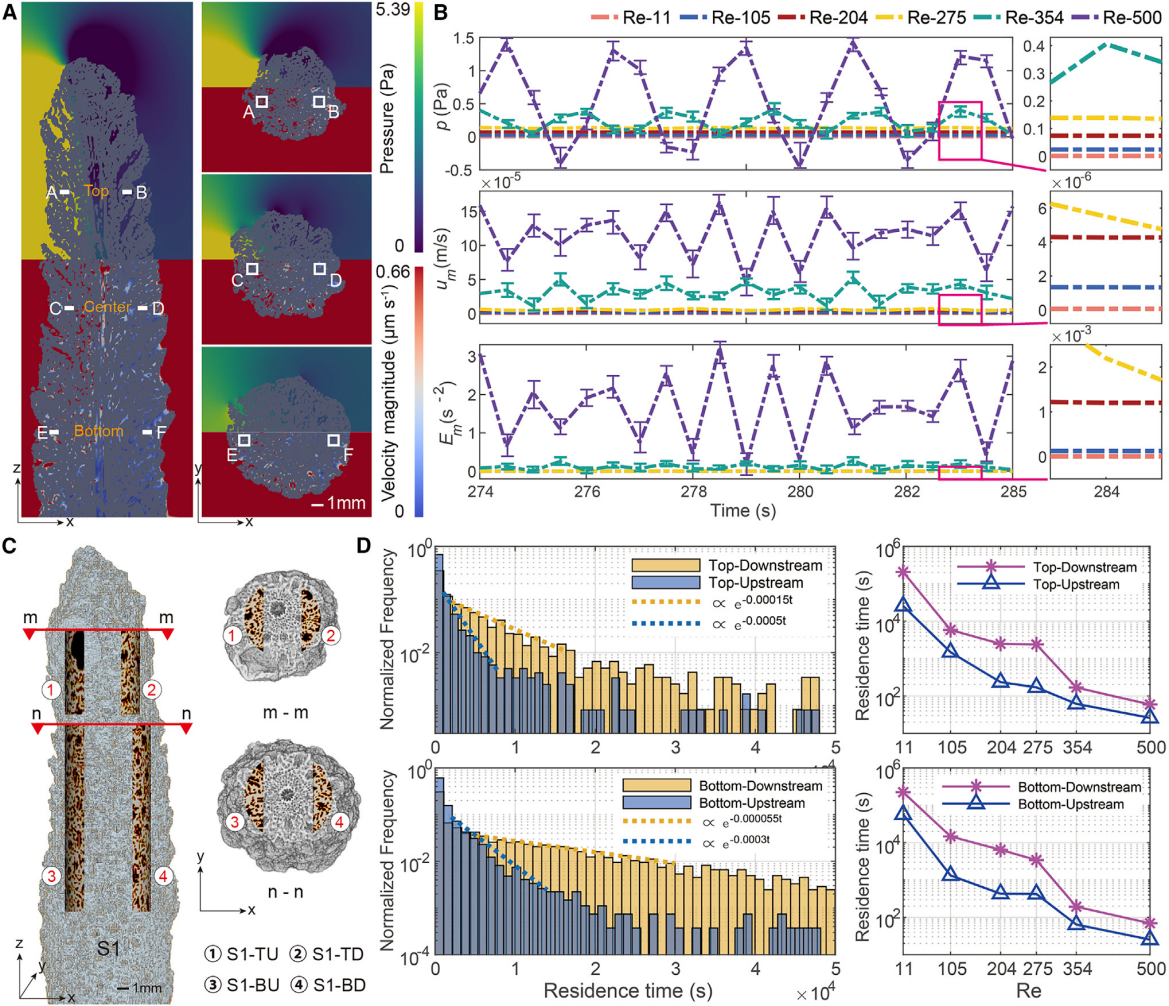
Asymmetric flow inside the skeleton structure (A) Slices of y = 255 (left), z = 90, 215, 340 (right), with pressure (upper) and velocity (lower) at Re = 115. A–F slices were selected (ranges and sizes shown in Table S4) at the top, center, and bottom to examine the difference between upstream and downstream. The color shows the magnitude of the pressure and velocity. Scale bar: 1 mm. (B) Average differences of pressure, velocity, and enstrophy between A and B, C and D, and E and F of simulations with increasing Re at the quasi-steady state. Error bars indicate standard errors of the mean. (C) Four sub-volumes were selected for analyzing the internal flow characteristics: ① S1-Top Upstream, ② S1-Top Downstream, ③ S1-Bottom Upstream, and ④ S1-Bottom Downstream (see Table S4 for further details). Scale bar: 1 mm. (D) Normalized statistical distribution of the particle’s residence time for the top (Figure 2D, left top) and bottom (Figure 2D, left bottom) section, showing the skeletal flow asymmetry, and the averaged residence times of 4 sections varying with increased Re (Figure 2D, right). Confidence level: 95% streamlines. Dashed lines show the concentration ratio of lower residence time frequency. “Normalized frequency” refers to occurrence frequency divided by the total one and the total frequency of 1 set of data (e.g., normalized frequency at top section) equals 1.

We first examined flow inside the interconnected radial cavities (radial corallites) around the central axis channel in comparison with the ambient flow outside and close to the skeleton surface. The variations of flow velocity, vorticity, and pressure (Figure 2A; Video S2) clearly show the asymmetry of water flow in the annular area inside the skeleton, i.e., the dominance of the upstream flow over the downstream flow. Similar flow asymmetry was found outside near the skeleton surface (Figure S8). Six slices (Figure 2A) inside the skeleton were selected to calculate the difference in pressure, velocity, and vorticity between the upstream and downstream at the quasi-steady state under different Reynolds numbers (Figure 2B). The differences remained positive, indicating a stronger flow condition in the upstream region compared with the downstream. Again, flow asymmetries inside and outside the skeleton show similarity for all flow parameters. Note that the relative differences (i.e., upstream value minus downstream value divided by the latter in Figure S8) indicate stability for the highest Re, signaling a steady regime where the flushing times align with the ambient flow. Positive enstrophy difference ratios also underscore quicker flushing times in upstream sections compared to downstream.

To further quantify the asymmetric flow and validate the observed differences in flushing times mentioned above, we divided the internal flow region into four sub-volumes: top ①② and bottom ③④ (Figure 2C) and released 120,000 tracer particles in each sub-volume from its geometric center area of a radius of 150 LBM cells to track the movement and compute the streamlines of all particles flowing with water. The time of each streamline flowing through the section was recorded to indicate the particle’s residence time in the sub-volume. Shorter residence time represents higher efficiency of flow and mass transport. The normalized frequency of the particle residence time in the upstream was significantly lower than that in the downstream for both the top and bottom sections in the large time value range but higher for short times (Figure 2D, left; Re = 115, as an example). The residence time of 95% main flow lines in the upstream section decreased with increasing Re (Figure 2D, right); the decline became gradual as Re increased under laminar flow conditions but intensified as the flow turned turbulent. Overall, the flow entered the skeleton structure through the radially interconnected corallites on the upstream side and subsequently diverted to the axial (vertical) flow in part with the rest remaining in the radial direction and exiting from the downstream side. These general flow paths result in the asymmetric radial flow with the upstream dominance.

### Dominant flow inside the central axial channel

**Figure 3:**
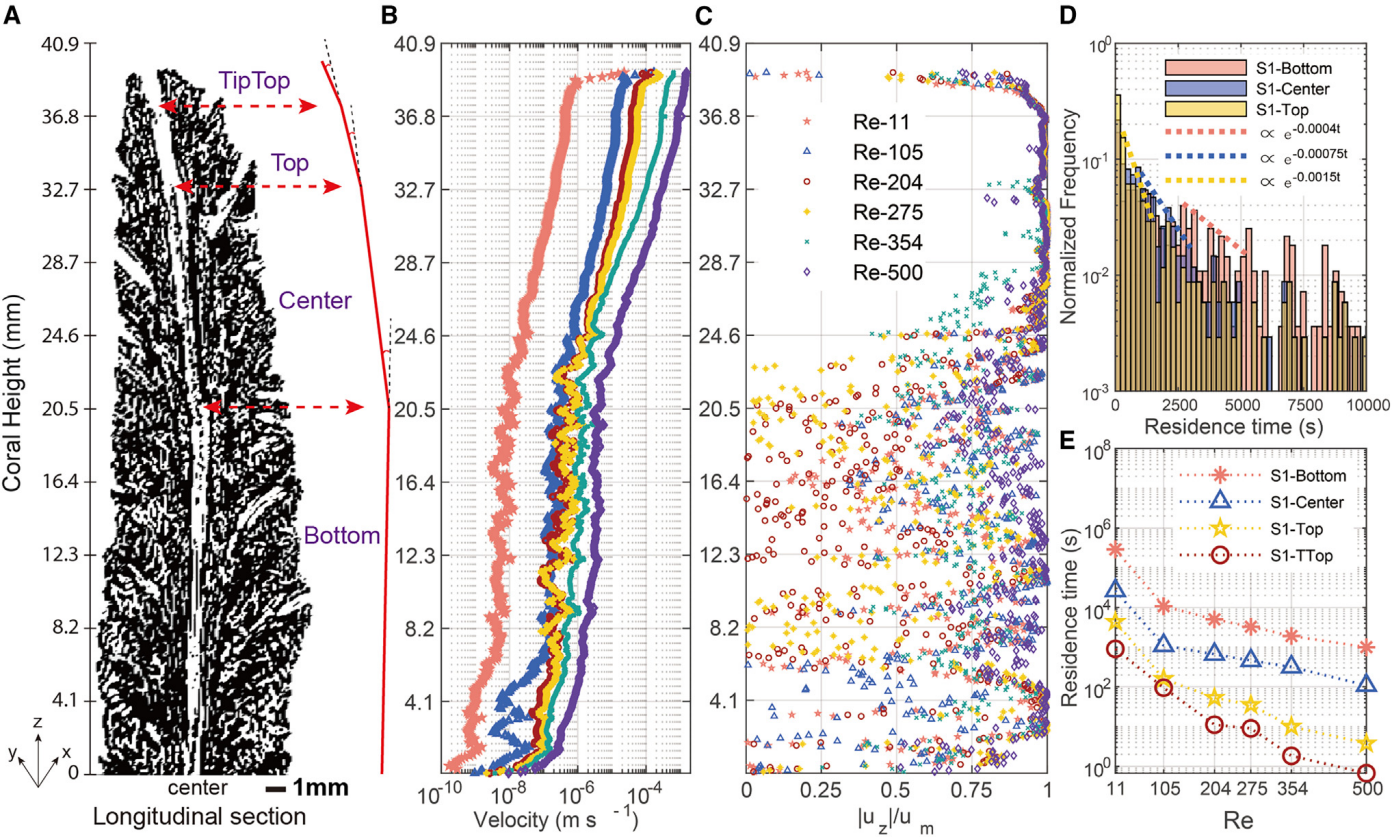
Characteristics of flow in different domains along the coral skeletal bending axial channel (A) Longitudinal section of S1 (angle of section see Figure S2A) shows the bending axial channel inside the *Acropora* coral center. Four parts (TipTop, Top, Center, and Bottom; further details in Table S4) are divided by the three break points. Scale bar: 1 mm. (B) Average flow velocity magnitude with different Re along the axial channel. (C) Ratio of the z direction velocity components ($u_{z}$) to the total velocity magnitude within the channel varies with increased Re. (D) Normalized statistical distribution of particle residence time for the top, center, and bottom sections of the skeletal axial channel. Confidence level: 95% streamlines. (E) The residence time of the 4 sections changes with increased Re. Confidence level: 95% streamlines.

The central axis of *A*. *pulchra* coral extends in the direction of the growth of the axial corallites located at the top of its branch. *Sample*.1 exhibited this interesting axial feature axis. The micro-CT scan results revealed slight bending at three inflection points on the central axis, dividing the axial channel into four parts: TipTop, Top, Center, and Bottom (Table S4; Figure 3A). The bending of the central axial channel at the three inflection points is subtle, but corresponds with the flow regime changes. From Top to TipTop, the axial flow dominated, with the average velocity magnitude increasing significantly upward along the axial direction (Figures 3B and 3C). From Center to Bottom, the axial velocity component gradually diminished and turned to horizontal (radial) flow (Figures 3B and 3C). The flow at the bottom of the central axis became dominated by the horizontal component. Similarly, through particle tracking (Figure 3D), the estimated residence time in the Top was found to be significantly shorter than that in the Bottom (Re = 115). The vorticity was also more intense in the coral top (Videos S6, S7, S8, S9, and S10), where the axial flow became more dominant with increased Re (Figures 3D and 3E). These flow characteristics remained largely the same in the case of S1R with the skeleton placed in a different horizontal orientation (rotated by 180°).

Video S6. The simulation process (274–285 s) of ambient vorticity in S1, S1F, S3 at Re = 105

Video S7. The simulation process (274–285 s) of ambient velocity in S2, S2R at Re = 24, 218, 431

Video S8. The simulation process (274–285 s) of ambient vorticity in S2, S2R at Re = 24, 218, 431

Video S9. The simulation process (274–285 s) of inner velocity in S2, S2R at Re = 24, 218, 431

Video S10. The simulation process (274–285 s) of inner vorticity in S2, S2R at Re = 24, 218, 431

### Increased vorticity on the surface bumps

**Figure 4:**
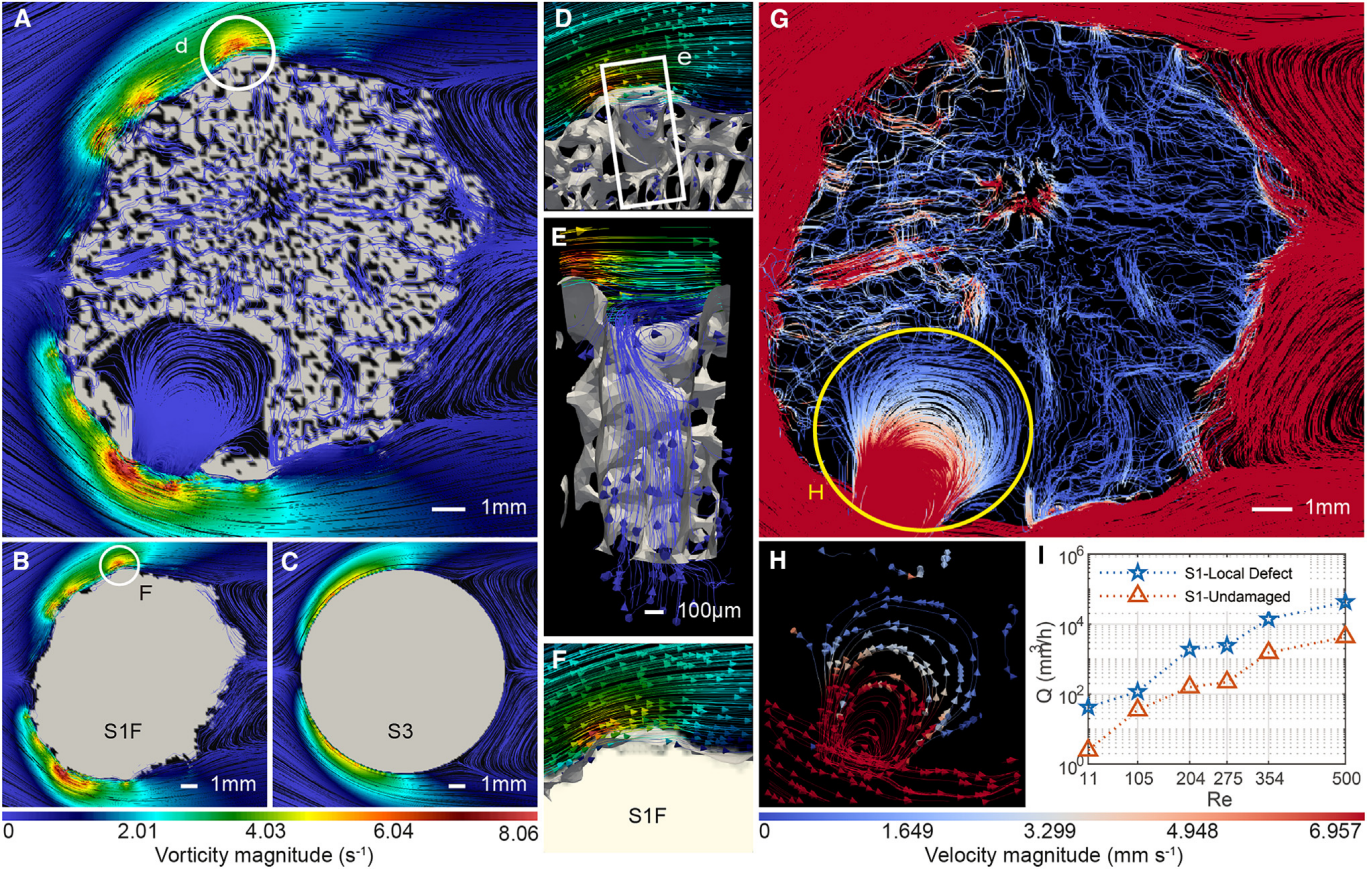
Vorticity, velocity, and flow flux within the surface bumps and interconnected calyx corallites (A–C) Vorticity increases due to the surface roughness and internal pore space and flow in S1 compared with S1F where the skeleton pores of S1 were filled (further details shown in Figure S2B) and S3 with a “smooth” cylinder (a solid cylinder model (height = 4 cm, diameter = 1.23 cm)). Scale bar: 1 mm. (D–F) Each corallite on the upstream side leads to an increase in vorticity. (D) shows an enlarged view of the section highlighted by the white circle in (A). The bump of the corallite generated the eddy, which was enhanced by the upward, spinning internal flow in the pore underneath as shown in (E) (scale bar: 100 μm.). (F) shows an enlarged view of the section highlighted by the white circle in (B). (G) The internal flow velocity in the case of S1 with velocity magnitude and direction is shown. Scale bar: 1 mm. (H) Local defect (yellow circle) with velocity magnitude and direction shown. (I) Flux comparison between the local defect and central undamaged sites (for details, see Table S9). Confidence level: 95% streamlines.

Each corallite, based on radial corallites, is a fundamental polyp growth unit^34^ and forms distinct bumps on the skeleton surface, increasing surface roughness.^35^ The increase of vorticity on the skeleton surface is closely related to the bumps. By comparing the hydrodynamic simulations among the cases of the natural coral skeleton (S1), the filled coral skeleton (S1F, which is S1 with the internal pores filled), and the smooth solid cylinder (S3, a solid cylinder with similar dimensions as the coral skeleton sample) as shown in Figures 4A–4C and Video S6, we found that the vorticity near the bumps on the side facing the coming flow were high according to the vorticity color threshold. This means that the corallite in which the polyps live produced significantly higher vorticity flow conditions on the skeleton’s surface. This vorticity creates an environment where nutrients are sucked in to support the polyp’s growth. Interconnected annular pore corallites led to the formation of vortices in the corallite (Figures 4D and 4E). The passive flow within the skeleton, induced by pressure gradients through the interaction of the skeleton with ambient seawater current, may play a significant role in the long-term adaptive growth mechanism of the living corals potentially facilitated by intercellular transport.^36^ This flow may contribute synergistically to active processes, such as the waving of the polyp’s tentacles.

It should be noted that the whole coral skeleton structure is not geometrically symmetrical along the central vertical axis. This asymmetry is evident by comparing S1 with its 180° rotated counterpart, S1R. Although turbulence was more prominent to occur in S1 (at the onset of Re = 354), all the flow features described previously remain the same in both S1 and S1R. Therefore, we propose that for coral skeletons devoid of soft tissues (polyp, coenosarc, etc.), despite the external skeleton geometry being asymmetrical, there are clear common flow characteristics, such as the vertical dominant flow, which may have developed through the co-evolution of coral tissue and skeleton to enhance coral growth. The simulations also showed larger internal flow and mass exchange rates (shorter particle residence time in the skeleton) with increasing Reynolds number.

### Erosion threat at the local defect

Coral skeleton structures and reefs are ideal habitats for polyps and other marine organisms,^37^ but they are not immune from attacks by natural enemies,^38^ resulting in defects. S1 had a non-negligible local defect caused by natural enemy gnawing tissues (polyps or periosteum) and skeleton.^38^ The absence of adequate coral tissue cover at the defect prevents calcification from occurring, especially when exposed to ocean acidification.^39^ We visualized the inner features (Figure S1-⑤) and flow conditions (Figures 4G and 4H) at the local defect (Figure 1A) and compared the flow rate (Figure 4I) with that in the undamaged central parts (Table S7). Much higher velocity and flux were found in the defect than elsewhere, which may impose a threat of local erosion to the skeleton.

### Acidification damage prediction in the future

We assessed and estimated this risk by using the data of simulated internal flows, based on two dissolution scenarios in year 2023 and 2100. Metabolic dissolution induced by localized production of protons from the respiration/decomposition of organic matter or microbioerosion by endoliths^40^ as acid-producing bioeroders^41^ is considered in addition to environmental dissolution^42^ caused by global acidification.^43^ In the first scenario, the localized proton production occurs outside the skeleton, e.g., reef sediments,^44^^,^^45^ which leads to the ingress/skeleton’s surface dissolution first, with the excessive proton transported inside by the internal flow. Because the dissolution rate is relatively high compared with the internal flow/transport rate, the excessive proton transported inside the skeleton reacts completely to dissolve the equivalent amount of CaCO_3_. In the second scenario, the localized proton production occurs inside the skeleton^41^ and maintains a locally stable pH level that is assumed lower than that of the ambient seawater. This leads to the skeleton in a dissolved state all along with pore water of elevated calcium cation concentration, producing a calcium efflux from which the amount and rate of CaCO_3_ dissolution can be estimated. Details of the analysis can be found in the STAR Methods section.

The results show relatively low dissolution rates of CaCO_3_ in the present situation, ranging from 3.08 × 10^−10^ to 5.53 × 10^−6^ mol m^−3^ s^−1^. These rates cause the skeleton porosity to increase annually by less than 1%, which is unlikely to reverse the net accretion of CaCO_3_ to affect coral growth. However, if the ocean acidification continues to intensify as predicted with the global ocean pH dropping to 7.87 by 2100,^46^ this, combined with additional acid from local respiration, will result in a significant increase of the CaCO_3_ dissolution rates, up to 64 times in the current level. We estimated that the porosity of the eroded skeleton will increase by about 50% only for one year in 2100, which is far higher than the estimation of a 20% average decline in coral reef density by the end of the 21st century due to ocean acidification alone.^26^

## Discussion

### Implications of internal flow characteristics for coral growth and stability

The porous space inside the *A*. *pulchra* skeleton provides a structural basis for material exchange between the skeleton’s interior and ambient seawater.^47^ The simulation results show that this deep exchange leads to complete flushing of the entire dead skeleton’s pore space, which can be completed on average within hours (Table S10). Especially for individual corallites—home of the polyps (Figures 4D and 4E)—high flow velocity significantly reduces the flushing time by a factor of 10 (Table S10). Previous studies on material transport for sustaining coral growth focused on the flow process within the boundary layer on the skeleton’s surface and its cellular tissue, i.e., the external transport pathway (Figure S9).^36^^,^^44^^,^^48^^,^^49^^,^^50^ For example, the supply of Ca^2+^ as a reactant for calcification and removal of H^+^ as a product is through this external transport pathway, from seawater through the tissue/polyp layer to the extracellular calcifying medium (ECM) where Ca^2+^ accumulation and skeletal structures are further formed.^51^^,^^52^ Here, we speculate that the internal flow system formed by the complex skeletal pore structure provides a possible alternative but neglected transport pathway for mass transport, that is, free calcium ions in the pores are directly and quickly transported and connected to the calcified site by the migration of internal water. As indicated by the relatively short flushing time and high rate of material exchange, the internal transport pathway may play a significant role in transporting materials to sustain the coral growth. Indeed, the internal flow characteristics revealed by the simulations appear to support or be consistent with the coral growth trends.

The simulations showed the asymmetric radial flow with upstream dominance regardless of the ambient flow direction. The question is whether such flow asymmetry based on the skeletons of dead corals would also lead to similar and more efficient material transport within living corals, making growth conditions more favorable upstream. Existing field experiments showed that when exposed to a unidirectional ambient flow for four months, the coral skeleton displayed asymmetric growth and morphology with bone thickening and even more new bumps on the upstream side.^53^ Therefore, we speculated that the observed radial skeleton morphology in the dominant growth region may be related to the asymmetric flow inside the skeleton in addition to the external flow asymmetry. What’s more, a gravity-detaching experiment showed that the corals grew in the opposite direction to the force on a horizontal surface,^54^ also indicating preferential growth in the upstream region. The asymmetric flow provides the upstream coral section with the mechanism for more efficient material exchange, including proton removal,^55^ and hence promotes the preferential coral growth on the upstream side. This is clearer from the comparison of residence times between the upstream and downstream areas (Figure 2D), shorter times indicating faster material exchange. In reality, unidirectional ambient flow in marine environment is unlikely to persist in any particular direction as far as coral branches are concerned. Across a coral colony, however, a strong directional component exists in skeletogenesis due to the differential growth rate; in particular, the outer coral branches and their side facing the external current experienced stronger flow and exchange with the ambient marine environment. Measurements showed that only about 15% of the ambient water flowing through a coral colony could reach the colony’s interior,^56^ where the flow rate decreases sharply. Although flow in the real marine environment is multi-directional, at the colony scale, the outskirt of the colony facing the external environmental current is expected to develop a relatively strong internal flow in the skeleton and become the growth-dominant region, similar to the outward growth of trees.^56^^,^^57^^,^^58^^,^^59^ This is consistent with the general trend of coral outward growth and expansion. Combining the insight from our simulations results with experimental observations of thicker and denser branches^60^ in coral colonies with high flow activities, we suggest that the adaptation of corals to flow conditions may be partly through the porous skeleton structure at the scale of the branch, in addition to channels and other features across colonies at the whole reef scale.^61^

The characteristic flow inside the coral central axial channel is divided into regions with different flow characteristics by its characteristic curved structure. Firstly, the increasingly strengthened upward flow from the top to tip-top section inside the characteristic corallite at the center may create optimal material transport for the apex polyps responsible for linear extension. The current here passively supported the important biological process such as photosynthesis and calcification in the coral polyp, helping to lead the direction of coral growth upward. It has been shown that the pore corallite in the top region is a fast calcification zone,^55^ with high ATP concentration,^62^ where the rapid flow exchange of nutrients and ions here provides material and energy conditions for rapid linear skeleton growth. However, due to the rapid growth, the density and elastic modulus^63^ of the top region are lower than that of other regions. In addition, in the living coral tissue, intensified water motion has been found to enhance photosynthesis and respiration of apical polyps.^55^ In contrast, when only considering the coral skeleton, the simulation results showed that horizontal flow dominates the central axial channel in the bottom section. This flow is fed by ambient water entering the skeleton from the upstream side and exiting downstream through the internal transport paths. We believe that this special internal transport mechanism, formed both by the skeleton structure and unidirectional ambient flow, may passively support the skeleton’s horizontal growth. These simulated flow characteristics are consistent with findings from a staining experiment on coral broken-off fragments, which showed that flow inside the central canal moves vertically at the apical tip but the flow direction in the bottom orientates sideways as the axial part of the base is more densely calcified.^64^ Such flow is favorable for the polyps with the annular radial distribution. The horizontal flow ensures outward growth and secondary accretion of the substructure,^55^ thickening the skeleton and increasing the density^65^ to fortify the stiffness and stability of the overall structure.^15^

The porous bump formed by each calyx (corallite) on the skeleton surface enhances the formation of vortices similar to the effects due to ciliary beating and tentacle waving by hard coral polyps^12^ and soft corals swinging around their bodies in response to waves^66^ to form the eddies. By reference experiments on particle capture, the trapping efficiency of a rough cylinder is 40%^67^ higher than that of a smooth one. We propose that this passive vortex caused by the rough skeleton surface has a similar function to the active enhancement vortex of coral tissue, which both increase material transport.

These effects lead to the expansion of the flow filtration area,^68^ enhancing larval feeding and trapping more nutrients, e.g., oxygen. Under high flow conditions, bumps are more likely to occur on the skeleton surface in nature.^69^ Again, we can see that the porous features of the skeleton structure directly induce flow conditions (the formation of vortices) that economize the energy for polyps to increase filter-feeding efficiency and hence growth.

### Potentially more serious threat from ocean acidification

Precipitation of CaCO_3_, which consumes significant amounts of energy, is an important part of the biological process of building the coral skeleton.^70^ Facing increasing ocean acidification with higher proton concentration in the seawater, this biochemical reaction may reverse and the coral skeleton becomes at risk of CaCO_3_ dissolution,^41^ threatening the delicate building block for coral growth. Previous studies on this threat have been focused on the reduction of the calcification efficiency and dissolution of CaCO_3_ from the skeleton surface^25^^,^^71^ and lacked consideration of the skeleton’s pore structure.^72^ The pore water flow pH inside the skeleton is lower than in the ocean. As a potential transport flow for bioeroders and microorganisms, it is likely to erode the coral skeleton from the inside both in dead and live corals. Here, we infer the potential risk to coral skeletons based on ocean acidity projections for 2023 and 2100, combined with flow simulations within dead coral skeletons.

We solved the transport equation for a pipe model with the flow information obtained from the simulations; please refer to the STAR Methods section. With this simple model we were able to produce an estimate of dissolution of the pore space due to acidification. We found a 45–51% increase in porosity (see Table S11; Figure S10), and as a consequence, the flexural rigidity of the skeleton will be reduced by almost 34%–46%, presenting a serious threat to the entire coral system, including the underlying skeleton and living corals. The reduction of the skeleton’s flexural rigidity makes it increasingly difficult for living corals to grow. Even worse, the coral branches, colonies, and reefs will struggle to stay intact both against ocean acidification, and increasingly more frequent and severe hurricanes and tsunamis due to climate change.^73^^,^^74^^,^^75^ The subsequent deepened water over coral reef further impact its ecosystem and the living condition for reef lagoon seagrass.^76^ Unfortunately, the actual situation may be even worse in that the net dissolution caused by diurnal/seasonal change in the difference between calcification/respiration of polyps^77^^,^^78^ and the decreased rates of calcification by polyps under more acidic conditions^79^^,^^80^ have not been taken into account. Under extreme ocean acidification conditions, the coral calcification is greatly reduced, while the dissolution caused by respiration is increased, which is detrimental to the CaCO_3_ net accretion for coral survival.^41^^,^^55^ The measured daily average dissolution rate of some reefs is far higher than our calculated values.^77^^,^^81^ Although we were aware of the risk of the CaCO_3_ dissolution under ocean acidification and the resulting threat to the coral eco-system, previous studies have been focused on the dissolution process on the skeleton surface or impact on coral polyps^25^^,^^71^^,^^82^; for example, in 2100, the perforate *Montipora* skeleton would lose on average 15 kg CaCO_3_ m^−2^y^−182^, and overlooked the internal direct erosion that is more detrimental because of the direct impact on the strength and stability of the basic coral reef-building unit. However, an opposite phenomenon has also garnered our attention. Acidification reduces the pH of surface water.^72^ Despite the simultaneous decrease in pH within calcified cells and at the coral tissue-skeleton interface, the pH gradient between the inside and outside of the cells increases,^83^ allowing the intermediate amorphous calcium carbonate (ACC) particles necessary for the calcification process to persist.^84^ Consequently, both the pH and aragonite saturation in the extracellular matrix (ECM) layer rise, rendering coral calcification less detrimental than anticipated.^83^^,^^85^ This correlation between calcium ions and calcification deposits in seawater^86^ strengthens our hypothesis that there may be an overlooked calcification pathway involving pore water flow within the coral skeleton and polyps. Calcium ions dissolved by acidification could rapidly re-enter the calcified layer through internal pore channels, maintaining a high calcium ion concentration. This hypothesis, however, requires further investigation for verification.

### Conclusions

In summary, our simulation results reveal the flow features inside the pure calcium carbonate coral skeleton, which are determined by the skeletal pore structure and found to be consistent with the growth and stability of *Acropora* coral. The results help us to explore the interconnection between the biotic and abiotic components of the coral ecosystem. Reef-building polyps create the whole framework, and the active water flow regulation involved in these biological tissues attached to the surface of the skeleton does, in the short term, profoundly affect the calcification of the entire coral skeletal structure. However, in the long term, the potential impact of pore water movement inside the skeleton on coral calcification couldn’t be ignored, as the flow ultimately entering the skeleton creates pressure gradients that assist in the calcification of coral polyps. Our simulation results suggest and highlight the effects of this pore flow and potential biological response mechanisms. The coral skeletons with specialized pore structures may facilitate environmental flow with internal flow and the material transport to allocate the limited resources for optimal coral growth. These two flows work synergistically to enhance material transport and promote coral growth. Such a passive adaptative growth mechanism of skeleton might have resulted from a long evolution process of the species in a relatively stable or slowly evolving marine environment. Starting from the passive flow transport inside the skeleton, our study verified the significance of internal flow’s existence in corals through simulation, providing a new way to further explore the adaptive growth mechanism of corals. However, under the rapid ocean acidification, we show here that the migration of this internal low-pH pore water may lead to a significant dissolution of CaCO_3_ inside the skeleton, reducing the coral’s ability to resist environmental risks, and imposing a serious threat to the coral ecosystem. The findings of the present study may have implications for a better understanding of the coral ecosystem and how it responds to climate change and ocean acidification. Such understanding is essential for making proper strategies and measures of coral conservation. The revealed ingenious skeleton structure has implications for future bionic designs of coral restoration plans and techniques.

### Limitations of the study

In this study, we examined water flow characteristics around the branching parts of *A*. *pulchra* corals. It is crucial to recognize that these findings do not necessarily extend to other parts of *Acropora* corals. By comparing different coral morphologies (encrusting, hemispherical, tabular, corymbose, and branching), we selected *Acropora* corals with distinct internal circulation characteristics. The investigation focused on unidirectional flows at medium to low velocities, limiting the applicability of our results to scenarios involving high flow velocities or multidirectional flows. Our simulation of the water flow environment considered only the interaction between the fluid and skeleton, omitting gases and insoluble solids, which could impact the broader applicability of our findings. Furthermore, the research concentrated on the effects of coral skeletal structures on water flow characteristics, neglecting the influence of coral polyps, adding another layer of limitation to our study.

## Resource availability

### Lead contact

Further information and requests for resources and reagents should be directed to and will be fulfilled by the lead contact, Sergio Andres Galindo Torres (s.torres@westlake.edu.cn).

### Materials availability

This study did not generate new materials.

### Data and code availability

• The data and code have been deposited at OSF: https://osf.io/6aw28/?view_only=d9ba39b5da3544859edc39c7924ba0ce and are publicly available as of the date of publication. Accession numbers are listed in the key resources table.
• Simulations are carried out using the LBM module of Mechsys at https://mechsys.nongnu.org/.
• Any additional information required to reanalyze the data reported in this working paper is available from the lead contact upon request.

## Acknowledgments

We thank Westlake University High-Performance Computing Center for computational support and Mechsys for its LBM module used for the simulations of this paper https://mechsys.nongnu.org/. We would like to thank Prof. Dixia Fan for guidance on flow related to coral; Zhuan Ge for early guidance of Matlab and Paraview use; Songkai Ren for early guidance of Matlab use; JinXin Liu for early code guidance; Zi Li for discussion on the simulation setup; Chen Yu for early assistance of CT image processing and ilastik use; Xiangbo Gao and Rongrong Tian for CT scanning of *Sample*.*2*; Ruipeng Li for discussion on flow characteristics; Siyuan Jing for beautification suggestions of Figure 1; and Lijun Cui, Xiaogang Chen, and Hangtong Li for the discussion on skeleton erosion. The aforementioned researchers are all from Westlake University. We would like to thank Jiawen Tao from Peking University for the data analysis assistance of Figure 2B-Em and Guohui Hu from State Grid Jiangxi Economic and Technology Research Institute for discussion and comments on the original manuscript. We would like to thank the 10.13039/501100001809Natural Science Foundation of China (grant no. 41976162) for funding support.

## Author contributions

Y.T., S.A.G.T., and L. Li. conceptualized and administered the project; S.A.G.T. and L. Li. supervised the study; L. Li. acquired the funding; Y.T. and L. Lei. performed CT and got 3D models; S.A.G.T. provided the LBM software Mechsys; L. Lei., P.Z., and S.A.G.T. provided visualization suggestions; P.Z., H.H., S.A.G.T., L. Lei., and L. Li. provided advice and discussion; Y.T. wrote the original manuscript; Y.T., P.Z., H.H., S.A.G.T., L. Li., and L. Lei. reviewed and edited the drafts.

## Declaration of interests

The authors declare no competing interests.

## STAR★Methods

### Key resources table

**Table:** 

REAGENT or RESOURCE	SOURCE	IDENTIFIER
**Deposited data**		

Open Science Framework	https://osf.io/6aw28/?view_only=d9ba39b5da3544859edc39c7924ba0ce	https://doi.org/10.17605/OSF.IO/6AW28

**Software and algorithms**		

MATLAB 2024b	https://matlab.mathworks.com	RRID:SCR_001622
Mechsys	https://mechsys.nongnu.org	N/A
Paraview	https://www.paraview.org	RRID:SCR_002516
ImageJ Fiji	https://imagej.net/imagej-wiki-static/Fiji	RRID:SCR_003070; RRID:SCR_002285
3D Slicer	https://www.slicer.org	RRID:SCR_005619

### Experimental model and study participant details

The coral skeleton samples were collected from a dead Acropora *pulchra* coral colony, provided by Guangxi Key Laboratory on the Study of Coral Reefs, sourced in the South China Sea.

### Method details

#### Reconstruction of acropora coral skeletal structure

The coral skeleton samples were collected from a dead A. *pulchra* coral reef of the South China Sea. The colony was immersed in 5% sodium hypochlorite for 24 h to remove all organic residues, then rinsed under running water for 12 h. It underwent ultrasonic cleaning in anhydrous ethanol and distilled water for 30 min each, and was dried at 33°C for 24 h. After ultrasonic cleaning and drying, the two coral branches cut from the colony, labeled as Sample.1 and Sample.2, were scanned using a micro CT-scan device (model number: Xradia 610 Versa) to obtain 3D images of the skeleton structure in a digitized form. The top of Sample.1 contained a local defect (caused by external disturbance, e.g., animal erosion).

We used the pixel classification module based on the interactive machine learning method in ilastik^87^ for image analysis to accurately segment the gray pixel values of the coral skeleton solid (calcium carbonate in the form of aragonite) and pores (solid assigned a value of 1, and pores 2) to obtain the three-dimensional geometric structure of the coral skeleton. Here, we focus on the hydrodynamic interaction between the coral skeleton and ambient flow, and thus neglect the density changes of the skeleton solid itself. The actual physical dimensions of the coral skeleton samples were determined: Sample.1 is about 4 cm high and 1.2 cm in diameter, while Sample.2 is about 2.4 cm tall and 1.1 cm in diameter.

The parameters and accuracy of the Micro-CT are shown in Table S1. Although a highly refined scanning sample is desirable, it would make the simulation overly costly in terms of the computational time. To save the computation cost while ensuring a sufficiently accurate representation of the skeleton structure, we reduced the simulation domain of Sample.1 and Sample.2 by four times in equal proportion in XYZ directions. Sample.1 with the drop location of the left coral in Figure 1B (labeled S1) is rotated clockwise about z axis by 180°to produce the simulation case of S1R so as to examine the condition with ambient water flow through the skeleton structure from another direction. Similarly, Sample.2 (right coral in Figure 1B, labeled S2) is rotated clockwise about z axis by 180°to make the case of S2R. The internal pore space of S1 is filled for S1F, which only retains the external structural roughness so that the water flow could only bypass the outside of the skeleton instead of entering it. In the same way, cases S1RF and S2RF are generated and simulated. Coral inner pores from Sample.1 were identified with image processing, the value of 1 was assigned to inner pores and combined with redefined skeleton 0 and external pore 2 only for further whole flux calculation inside the coral.

Based on the binary data of the coral skeleton and pores, the porosity of the skeleton as a whole or in part can be calculated directly. Here, the porosity n of *Sample.1* and the specific field is

(Equation 1) $$\begin{array}{c} n_{S1}=\frac{S1\left(2\right)-S1F\left(2\right)}{S1\left(1\right)}\text{,} \end{array}$$

(Equation 2) $$\begin{array}{c} n_{specific\;range}=\frac{S_{sr}\left(2\right)}{S_{sr}\left(1\right)}\text{.} \end{array}$$

S1(2) is the pores of the whole simulation domain on *Sample.1* (both the pores inside the coral skeleton and the ambient environment (non-solid part) with the value equal to 2), S1(1) is the coral skeleton with value equal to 1, and S1F(2) is the ambient flow environment (non-solid part, value equals 2) on the simulation of S1F (the coral pores of *Sample.1* were filled). The specific range is chosen for more detailed analysis that only refers to coral skeleton (solid part) and inner pores (non-solid part).

#### The Lattice Boltzmann Method for flow simulation

The LBM^88^ is an efficient computational fluid dynamics method based on physical principles at a mesoscopic scale.^89^ It is particularly beneficial when dealing with complex and irregular geometries and adapts well to the flow inside the coral of the complicated porous structure.

For the present study, we use the D3Q19 scheme^90^ to divide the space into a cubic domain where each cell has a set of probability distribution functions $f_{i}$ that follow the lattice Boltzmann equation,

(Equation 3) $$\begin{array}{c} f_{i}\left(x+e_{i}\delta_{t},t+\delta_{t}\right)-f_{i}\left(x,t\right)=\frac{1}{\tau }\left[{f_{i}}^{eq}\left(x,t\right)-f_{i}\left(x,t\right)\right]\text{,} \end{array}$$

(Equation 4) $$\begin{array}{c} {f_{i}}^{eq}=\omega_{i}\rho \left(1+3\frac{e_{i}\cdot u}{C^{2}}+\frac{9{\left(e_{i}\cdot u\right)}^{2}}{2C^{4}}-\frac{3u^{2}}{2C^{2}}\right)\text{,} \end{array}$$

where $f_{i}$ represents the probability distribution function of fluid particles that move along with discrete velocities $e_{i}$ at position x. $\delta_{t}$ is the time step, and τ is the characteristic relaxation time which is related to fluid viscosity. The right side of the equation is the collision operator with Bhatnagar-Gross-Krook (BGK) model and ${f_{i}}^{eq}$ is the equilibrium distribution function.

The density ρ and velocity u at each cell x can be determined by

(Equation 5) $$\begin{array}{c} \rho \left(x\right)=\sum_{i=0}^{18}f_{i}\left(x\right)\text{,} \end{array}$$

(Equation 6) $$\begin{array}{c} u\left(x\right)=\frac{\sum_{i=0}^{18}f_{i}\left(x\right)e_{i}}{\rho \left(x\right)}\text{.} \end{array}$$

The ideal gas law gives the relationship between the density and pressure for this model

(Equation 7) $$\begin{array}{c} p=\frac{C^{2}\rho }{3}\text{,} \end{array}$$

where $C=\delta_{l}/\delta_{t}$ is a characteristic lattice velocity defined by the grid spacing $\delta_{l}$.

The simulation domain of *Sample.1* (with the local defect), in a cubic shape, comprises 499×510×988 LBM cells. *Sample.2* has 629×638×739cells, and *Sample.3* has 499×510×988 cells.

The boundary conditions applied in the simulation as shown in Figure S4 are as follows.

(1) Inflow boundary: the velocity boundary with open channel velocity profile;
(2) Outflow boundary: the boundary with a constant pressure condition;
(3) Transverse side boundaries: periodic boundaries;
(4) Top boundary: free-slip boundary condition to simulate the free sea level;
(5) Bottom boundary: no-slip boundary to mimic the seabed environment.

These boundary conditions simulate the interaction of a submerged coral branch grown from the seabed. The maximum flow velocity at the inlet was increased to cover different ambient flow conditions of low and medium velocity^53^ that may be encountered in reality, corresponding to a range of Reynolds numbers: for S1, the preset physical velocities are u = 0.1, 0.9, 1.7, 2.3, 3.0, 4.2 cm/s giving Re = 9, 81, 153, 210, 270, 378; for S2 u = 0.3, 2.4, 4.8 cm/s and Re = 19,163,327; and for S3 u = 0.1, 4.2 cm/s and Re = 9, 378). The definition of Reynolds number is given as follows,

(Equation 8) $$\begin{array}{c} Re=\frac{|u_{inlet}|\cdot W}{\nu }\text{,} \end{array}$$

where $u_{inlet}$ is the maximum velocity of inlet ambient flow, W is the approximate diameter of the coral skeleton (*Sample.1* is 1.2 cm, *Sample.2* is 0.9 cm, *Sample.3* is 1.2 cm), ν is the water kinematic viscosity ($1.0\times 10^{6}m^{2}s^{-1}$). Detailed parameters and actual measured values (measured physical velocity of S1: u = 0.09, 0.85, 1.66, 2.24, 2.89, 4.07 cm/s; S2: u = 0.26, 2.38, 4.72 cm/s; S3: u = 0.09, 4.07 cm/s; measured Reynolds number of S1: Re = 11, 105, 204,275, 354, 500; S2: Re = 24, 218, 431; S3: Re = 11, 500) are shown in Table S2 as the measured simulation conditions finally applied on the samples slightly differ from the preset ones (errors), and a conversion factor of LBM and physical values are shown in Extended Table S3.

The simulations were run on the supercomputing facilities (HPC Linux Cluster) at Westlake University. The simulations on *Sample.1*, *Sample.2*, and *Sample.3* involved 28 tasks of the whole model and 64 threads for each task. The simulation duration of *Sample.1* and *Sample.3* is about 558 s of physical time, and that of *Sample.2* is 109 s. The LBM time steps were controlled by the physical time (e.g., preset Re = 9 in *Sample.1*, LBM steps = 500,000), as shown in Table S2 for details. The simulations on the two samples presented here required about 20,000 CPU hours, resulting in 275 terabytes of raw data.

ParaView and MATLAB were used to analyze the simulation results further. Enstrophy is used to measure the strength of vorticity, which is defined as

(Equation 9) $$\begin{array}{c} E=\frac{{\left|\omega \right|}^{2}}{2}=\frac{{\left|\nabla \times u\right|}^{2}}{2}\text{.} \end{array}$$

Particles were released to track the flow, with the streamline computed to trace the particle’s trajectory of movement with the flow. The average particle residence time within the specific selected skeleton section was calculated based on all the streamlines that go through the section, i.e.,

(Equation 10) $$\begin{array}{c} \bar{t}=\frac{\sum \frac{\Delta l}{\Delta u}}{n_{streamline}}=\frac{\sum_{i}^{n}\frac{\sqrt{{\left(l_{ix}-l_{\left(i+1\right)x}\right)}^{2}+{\left(l_{iy}-l_{\left(i+1\right)y}\right)}^{2}+{\left(l_{iz}-l_{\left(i+1\right)z}\right)}^{2}}}{\sqrt{{u_{ix}}^{2}+{u_{iy}}^{2}+{u_{iz}}^{2}}}}{n_{streamline}}\text{,} \end{array}$$

Where $n_{streamline}$ is the number of streamlines flowing through the specific section, $l_{\left(i+1\right)x,y,z}{-l}_{\left(i\right)x,y,z}$ is the difference between position components defining the streamline and $u_{\left(i\right)x,y,z}$ is the velocity component at that point obtained by interpolation from the the LBM cells. Further details are given in Tables S4 and S9.

The average volume flow flux can then be estimated by the following equation,

(Equation 11) $$\begin{array}{c} Q=\frac{Inner\;pores\;Volume}{\bar{t}}\text{.} \end{array}$$

#### Assessment of the effect of ocean acidification on the coral skeleton

Water flowing into the skeleton is under-saturated with the Calcium cation and in a relatively low pH condition owing to the CO_2_ production by remineralization of organic matters in the reef, sediment, and even skeleton microenvironments.^26^^,^^41^ Thus, the biological and chemical dissolution of calcium carbonate (aragonite) may occur in the skeleton,

(Equation 12) $$\begin{array}{c} {CaCO}_{3}+{{CH}_{2}O+O}_{2}\to {Ca}^{2+}+2{{HCO}_{3}}^{-}\text{,} \end{array}$$

(Equation 13) $$\begin{array}{c} {CaCO}_{3}+H^{+}\to {Ca}^{2+}+{{HCO}_{3}}^{-}\text{.} \end{array}$$

The dissolution of aragonite depends on the proton concentration and so is exacerbated by ocean acidification.^41^ By introducing an equivalent homogeneous calcium carbonate tube to mimic the coral branch with an outer diameter of $R_{O}$, an inner diameter of $R_{I}$ and a length of H as shown in Extended data Figure S10, we can simulate the transport and reaction of the proton, and dissolution of aragonite in a simplified way (assume the reaction equilibrium of the aragonite dissolution immediately reached and a closed system; assume the skeleton without any coral polyps attached to). Two scenarios were considered.

##### Scenario 1

If the acid is produced outside (sediment layer)^44^^,^^45^ and gets transported into the tube by the flow. we assumed enough protons in the acid flow of the entrance. When coming across the skeleton aragonite, the dissolution of calcium carbonate reacts directly and fast till the protons run out. The dissolution rate $r_{{CaCO}_{3}}$ (CaCO_3_ mol m^−3^s^−1^), also the proton consumption rate $r_{H^{+}}$ (H^+^ mol m^−3^s^−1^), is related to the concentration of proton $C_{\left[H^{+}\right]}$ (mol m^−3^) governed by the transport-reaction equation as follows,

(Equation 14) $$\begin{array}{c} -r_{{CaCO}_{3}}=-r_{H^{+}}=\frac{\partial C_{\left[H^{+}\right]}}{\partial t}+u\frac{\partial C_{\left[H^{+}\right]}}{\partial x}\text{.} \end{array}$$

where we assumed a one-dimensional flow of a constant velocity u. Diffusion is a weak transport process and is thus neglected. Based on the data of Morse,^91^ the dependence of the aragonite dissolution rate r (mol m^−3^s^−1^) on the proton concentration was determined through fitting (with a fit goodness R^2^ = 0.98), $-r_{H^{+}}=2.5\times 10^{-7}\left(e^{1.98\times 10^{5}C\left[H^{+}\right]}-1\right)$.

With this fitted relationship and the transport equation, an analytical solution can be found by the method of characteristics. Interestingly, the method of characteristics offers a time-independent solution for a given constant proton concentration at the inlet boundary (prior to entering the skeleton),

(Equation 15) $$\begin{array}{c} C\left[H^{+}\right]=-\frac{\ln\left(1-e^{-\frac{0.0496x}{u}}\left(1-e^{-1.98\times 10^{5}C\left[H^{+},x=0\right]}\right)\right)}{1.98\times 10^{5}}\text{.} \end{array}$$

This formula shows a very rapid decrease in the concentration of protons near the entrance of the tube if the pore water flow velocity is slow as it is in our case. Regardless of this close-form solution, the average dissolution rate $r_{ad}$ across the tube is given by

(Equation 16) $$\begin{array}{c} r_{oe}=\int_{0}^{H}r_{H^{+}}dx=-\frac{1}{H}\int_{0}^{H}u\frac{\partial C_{\left[H^{+}\right]}}{\partial x}dx=-\frac{u}{H}\left(C_{\left[H^{+},x=H\right]}-C_{\left[H^{+},x=0\right]}\right)\text{.} \end{array}$$

where the transport equation and the time-independence nature of its analytical solution have been used to evaluate the averaging integral. Note that the concentration of protons is related to pH, $C_{\left[H^{+}\right]}=10^{3-pH}$ (mol m^−3^). Since the reaction happens very quickly, the protons at or very near the pipe entrance are consumed in large quantities and so $C_{\left[H^{+},x=H\right]}=0$. Hence, the average reaction rate $r_{oe}$ (mol m^−3^ s^−1^) caused by outside acidic environment should be

(Equation 17) $$\begin{array}{c} r_{oe}=\frac{u}{H}\left[10^{3-pH\left(x=0\right)}\right]=\frac{Q}{H\pi {R_{I}}^{2}}\left[10^{3-pH\left(x=0\right)}\right]\text{.} \end{array}$$

##### Scenario 2

If the skeleton is always in a relatively low pH microenvironment due to the assuming prolonged biochemical reactions in the habitat system,^41^^,^^92^ protons inside the aragonite tube will react continuously. The dissolution rate $r_{ie}$ can then be regarded as relatively constant as given by Equation 15 with the low constant pH value. When coming flow from the ingress goes through the tube, the dissolved calcium cations will expel out following the flow (assuming pH difference between inside and outside the tube is zero). See Table S11 for further details.

In both scenarios, once the dissolution rate is determined, the amount of internal dissolved aragonite can be calculated as well as the enlarged pore volume $V_{skeleton}$ over time as follows,

(Equation 18) $$\begin{array}{c} V_{skeleton}\left(t\right)=\pi \left({R_{O}}^{2}-{R_{I}\left(t\right)}^{2}\right)H\text{,} \end{array}$$

(Equation 19) $$\begin{array}{c} R_{I}\left(t\right)={R_{I\;}\left(0\right)e^{\frac{r_{oe,ie}M}{2\rho }t}\approx R}_{I\;}\left(0\right)\left(1+\frac{r_{oe,ie}M}{2\rho }t\right)\text{,} \end{array}$$

where RI is the eroded inner radius, ρ is the skeleton particle density (here we use 2.7 g/cm^3^), M is the molar mass of CaCO_3_ (100 g/mol), and the linear approximation in Equation 19 is valid since $\frac{r_{oe,ie}M}{2\rho }t\ll 1$ (even for one century the value of this factor is on the order of 10^−2^).

The porosity change ratio N (%) can then be estimated,

(Equation 20) $$N_{oe,ie}=\frac{\Delta n_{oe,ie}}{n_{0}}\times 100\%=\frac{\frac{V_{skeleton}\left(0\right)-V_{skeleton}\left(t\right)}{V_{total}}}{n_{0}}\times 100\%=\frac{\frac{{R_{I}\left(0\right)}^{2}}{{R_{O}}^{2}}\left(\frac{r_{oe,ie}Mt}{\rho }+{\left(\frac{r_{oe,ie}Mt}{2\rho }\right)}^{2}\right)}{n_{0}}\times 100\%\text{,}$$

where $\Delta n_{oe,ie}$ is the porosity change of the two conditions and $n_{0}$ is the coral skeleton’s initial porosity. We compared the porosity change ratio of *Sample.1* and *Sample.2* over one year nowadays (2023) and in 2100 to evaluate the extent of dissolution damage on the coral skeleton due to ocean acidification (see Table S11).

#### Coral mechanical resilience

To further quantify the damage of the skeleton’s dissolution by this acidic tube conceptual model, we computed the reduction of flexural rigidity G (%),

(Equation 21) $$\begin{array}{c} G=\frac{\Delta EI}{EI}\times 100\%\propto \frac{{A_{skeleton,t=0}}^{2}-{A_{skeleton,t}}^{2}}{{A_{skeleton,t=0}}^{2}}\times 100\%\text{,} \end{array}$$

Where $A_{skeleton,t=0}$ is the area of the inner tube at the start point, and $A_{skeleton,t}$ is the ending time. According to the measured data of the branching coral skeleton,^93^ the relationship between 2D (planar total surface) and 3D (CT volume) metrics of the coral skeleton is fitting as log(volume)=1.4728log(area)−1.5275 ($R^{2}=0.9214$) (see Table S11).

$Q_{\text{whole}}$ is the flux inside the coral pores (also choose about 95% with different Reynolds numbers, $V_{skeleton}\left(t\right)$ related to porosity change is the skeleton dissolution volume through a whole year at 2023 and 2100, $V_{skeleton,t=0}$ is the initial skeleton’s volume. Detailed calculations of G are as follows:

(Equation 22) $$\begin{array}{c} G\propto \frac{{V_{skeleton,t=0}}^{1.358}-{V_{skeleton}\left(t\right)}^{1.358}}{{V_{skeleton,t=0}}^{1.358}}\times 100\%\text{.} \end{array}$$

#### Ilastik

Ilastik is a machine learning-based computer vision processing tool. Its functions include fast universal feature extraction, powerful nonlinear classifier, probabilistic graph model, and solver. Complete complex image processing tasks with simple and intuitive operations without requiring the user to have deep computing expertise. Ilastik provides predefined workflows covering applications such as image segmentation, object classification, counting, and tracking. Ilastik enables algorithmic customization of specific datasets through sparse training data based on user-defined labels. Then, the system processes the images in batches according to the training results and assigns different pixels to different labels defined.

### Quantification and statistical analysis

The data of Figures 1, 2, 3, and 4 are from Sample.1 and presented as mean ± standard deviation (SD). The quantification data of the tested groups Sample.1R, Sample.2, and Sample.2R are in supplemental information. MATLAB and Paraview were used for all analyses.
