# Supplementary material for: Internal hydrodynamics within the skeleton of Acropora pulchra coral
Source: iScience. 2025 Jan 4;28(2):111742. doi: 10.1016/j.isci.2025.111742 (PMC11804786; doi:10.1016/j.isci.2025.111742)
Supplement: Document S1. Figures S1–S10 and Tables S1–S11 [file mmc1.pdf]

iScience, Volume *28*

Supplemental information

Internal hydrodynamics within the skeleton of *Acropora pulchra* coral

Yanmei Tian, Pei Zhang, Hui Huang, Liang Lei, Sergio Andres Galindo Torres, and Ling Li

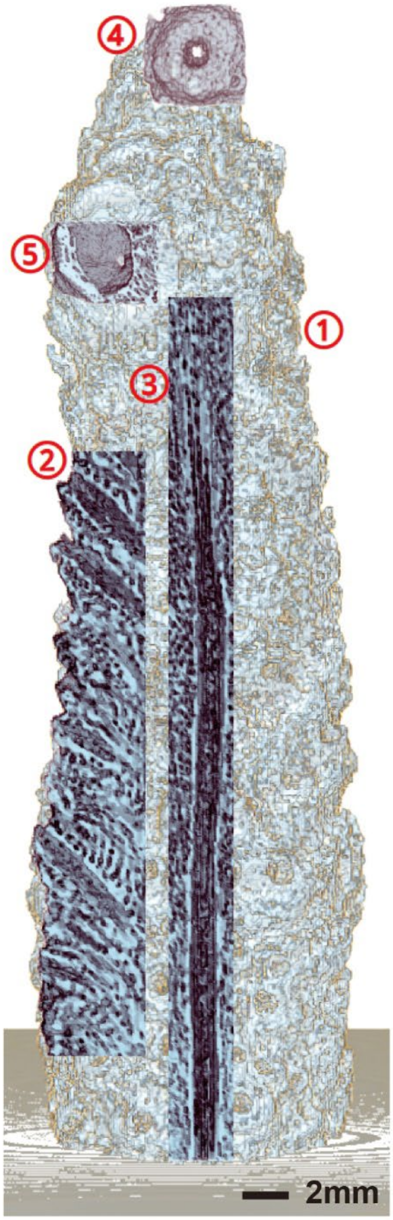

Fig. S1 | Features of skeleton subdivisional structures. (1) rough surface, (2) radial annular cavities, (3) central axial channel, (4) top calyx, and (5) local defect. The features are shown in Paraview, and features from ② to ⑤ are the 3D perspective of internal slices from ParaView. Scale bars: 2mm.

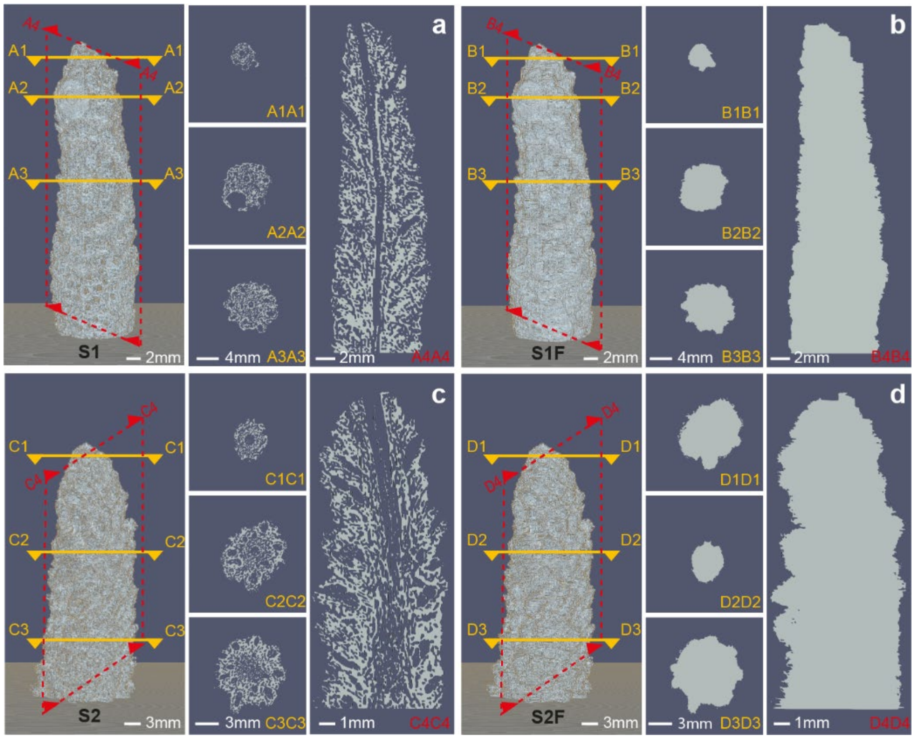

Fig. S2 | Cross and longitudinal sections of the two samples. a S1 and its four slices. A1A1 is z = 460, A2A2 is z = 380, A3A3 is z = 250, A4A4 is an oblique slice with origin $[x, y, z]$ = [250, 250, 494] and normal = [-1, -1, 0]. **b** S1F and its four slices. S1F is a simulated sample where the internal pores of S1 are filled. **c** S2 and its four slices. C1C1 is z = 400, C2C2 is z = 250, C3C3 is 100, C4C4 is an oblique section with origin $[x, y, z]$ = [330, 330, 369.5] and normal = [2, -1, 0]. **d** S1F and its four slices. S2F is a simulated sample where the internal pores of S1 are filled.

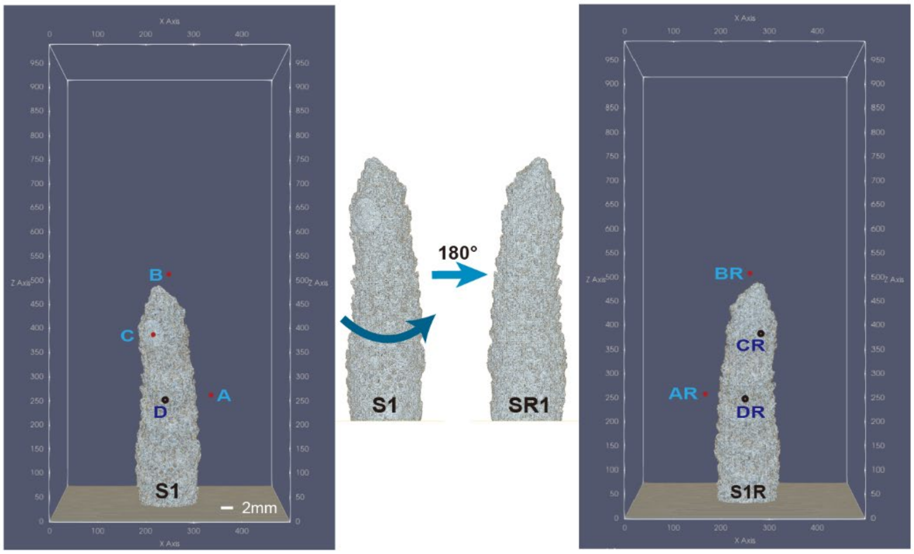

Fig. S3 | Four probes to monitor the state of flow condition. For S1, A donates the downstream region outside the coral skeleton (350, 255, 255), B represents the top area outside the skeleton (250, 255, 510), C signifies a local defect (250, 250, 250), and D indicates a typical point inside the skeleton (210, 210, 375). Rotating S1 around the z-axis by 180 degrees results in S1R. As for S1R, AR stands for the upstream region outside the coral skeleton (150, 256, 255), BR signifies the top area outside the skeleton (250, 256, 510), CR denotes a local defect (250, 261, 250), and DR indicates a typical point inside the skeleton (290, 301, 375). Scale bars: 2mm.

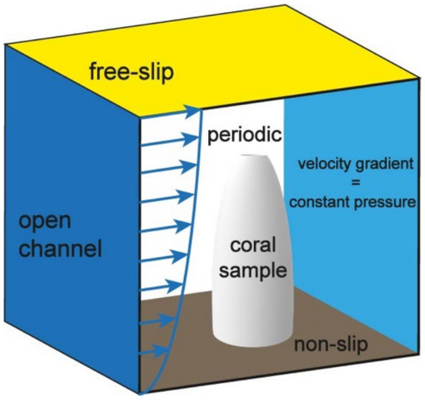

Fig. S4 | Details of boundary conditions applied in the simulations. The coral sample is positioned at the center of the simulation domain and the boundaries of the simulation domain are as follows:
Inflow boundary: the velocity boundary with the velocity profile over the depth specified;
Outflow boundary: the boundary with a constant pressure condition;
Transverse side boundaries: periodic boundaries;
Top boundary: free-slip boundary condition to simulate the free sea level;
Bottom boundary: no-slip boundary to mimic the seabed environment.

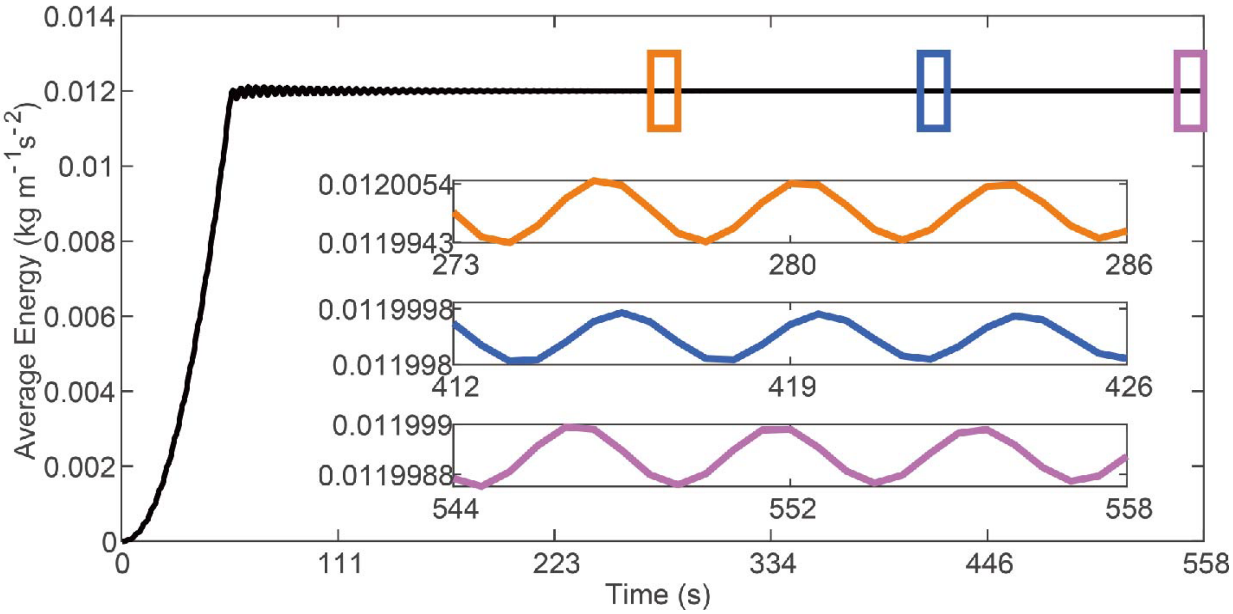

Fig. S5 | Average Energy of S1 at Re = 105 during the simulation process (0-558s). The orange rectangle represents the simulation time period from 273 to 286s, the blue one spans from 412 to 426 s, and the purple one covers the interval from 544 to 558 s. The simulation reached a quasi-steady state step after about 111s. To efficiently monitor the entire process, three distinct periods were selected. Notably, the data line between 273 and 558 seconds exhibits a state of near stability. We have chosen to concentrate our further analysis on the time periods corresponding to the red line, with the intention of conserving computing resources, assuming the rest of the simulation also maintains a quasi-steady state.

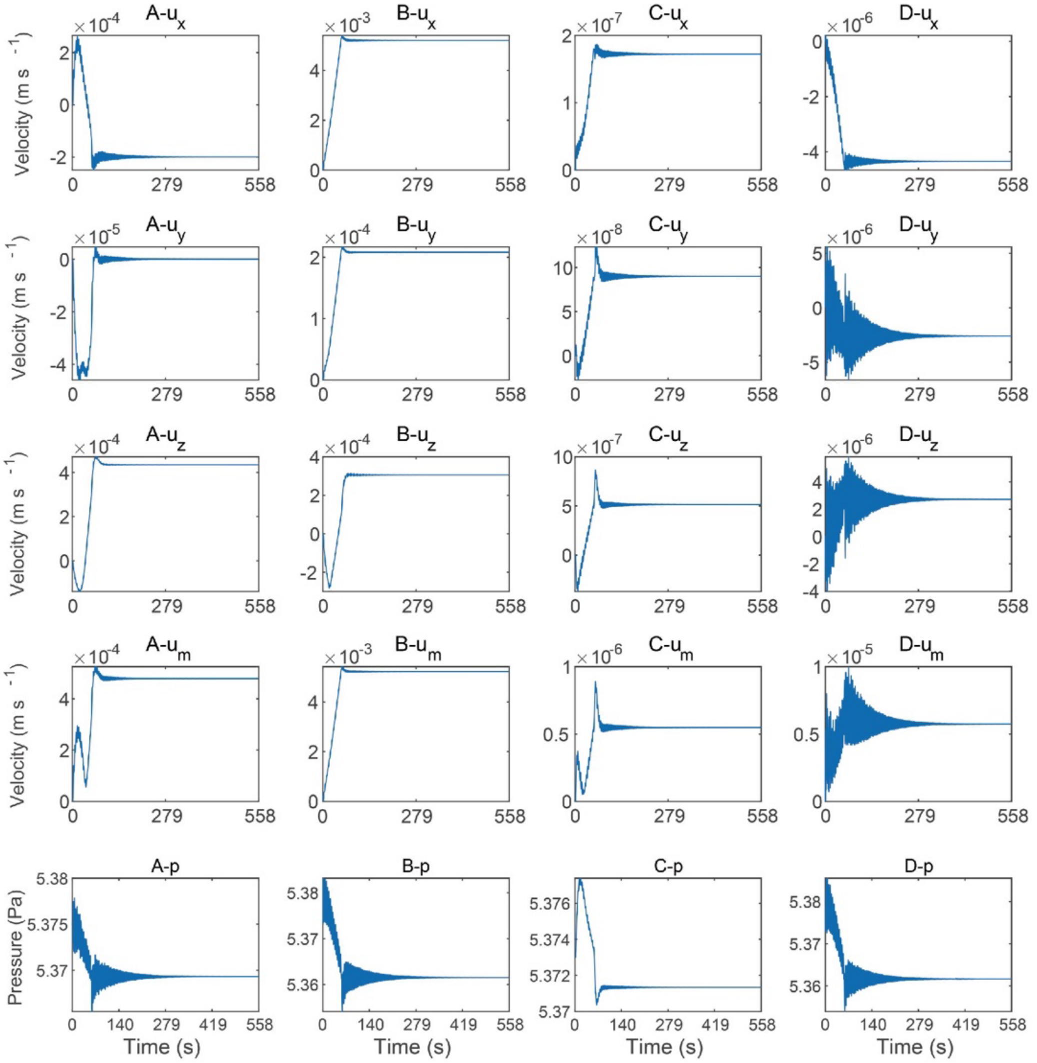

Fig. S6 | Velocity of four probes in Fig. S3a at Re = 105 during the simulation process (0-558s) in S1. Points A and B are situated outside the coral skeleton, with A downstream and B at the top of the skeleton. Points C and D are positioned within the skeleton, where C marks a local defect, and D is located internally within the structure. Details see Fig. S3a. The flow velocity and pressure at the designated points gradually stabilize over the course of the simulation. U_x, U_y, and U_z is the velocity component, U_m is the velocity magnitude, and p is the pressure.

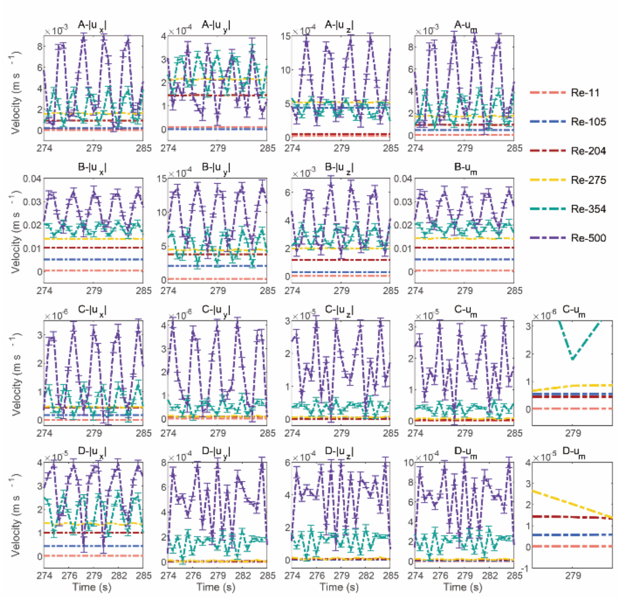

Fig. S7-1 | Absolute velocity at the four probes for Re = 11, 105, 204, 275, 354, 500 (274 – 285 s) in S1. Under various Reynolds number conditions, the absolute values of flow velocity and pressure at monitoring points A, B, C, and D (Fig. S3a) in the sample gradually stabilize over time, reaching a steady state (or attainment of steady-state averages). $|U_x|$, $|U_y|$, and $|U_z|$ are the absolute velocity components, while $|U_m|$ is the the velocity magnitude. The time span from 274 to 285 seconds is characterized as a quasi-steady state, as illustrated in Fig. S5. Error bars indicate standard errors of the mean.

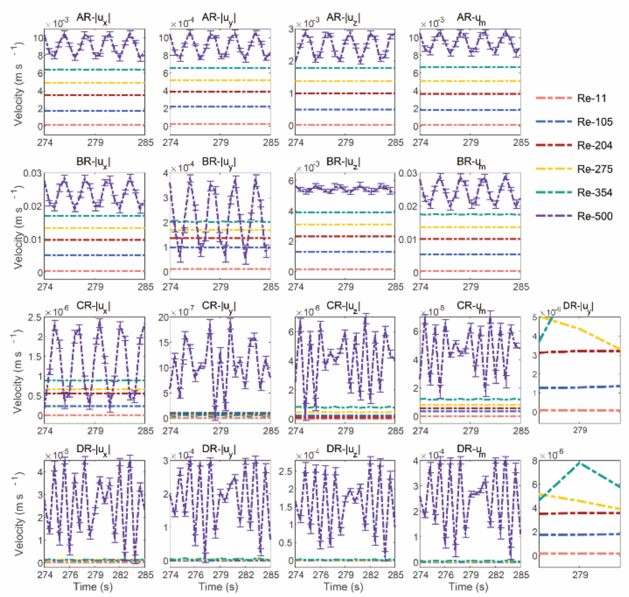

Fig. S7-2 | Absolute velocity at four probes for Re = 11, 105, 204, 275, 354, 500 (274 – 285 s) in S1R. Under various Reynolds number conditions, the absolute values of flow velocity and pressure at monitoring points AR, BR, CR, and DR (Fig. S3c) in the sample gradually stabilize over time, reaching a steady state (or attainment of steady-state averages). $|U_x|$, $|U_y|$, and $|U_z|$

are the absolute velocity components, while $|U_m|$ is the the velocity magnitude. Error bars indicate standard errors of the mean.

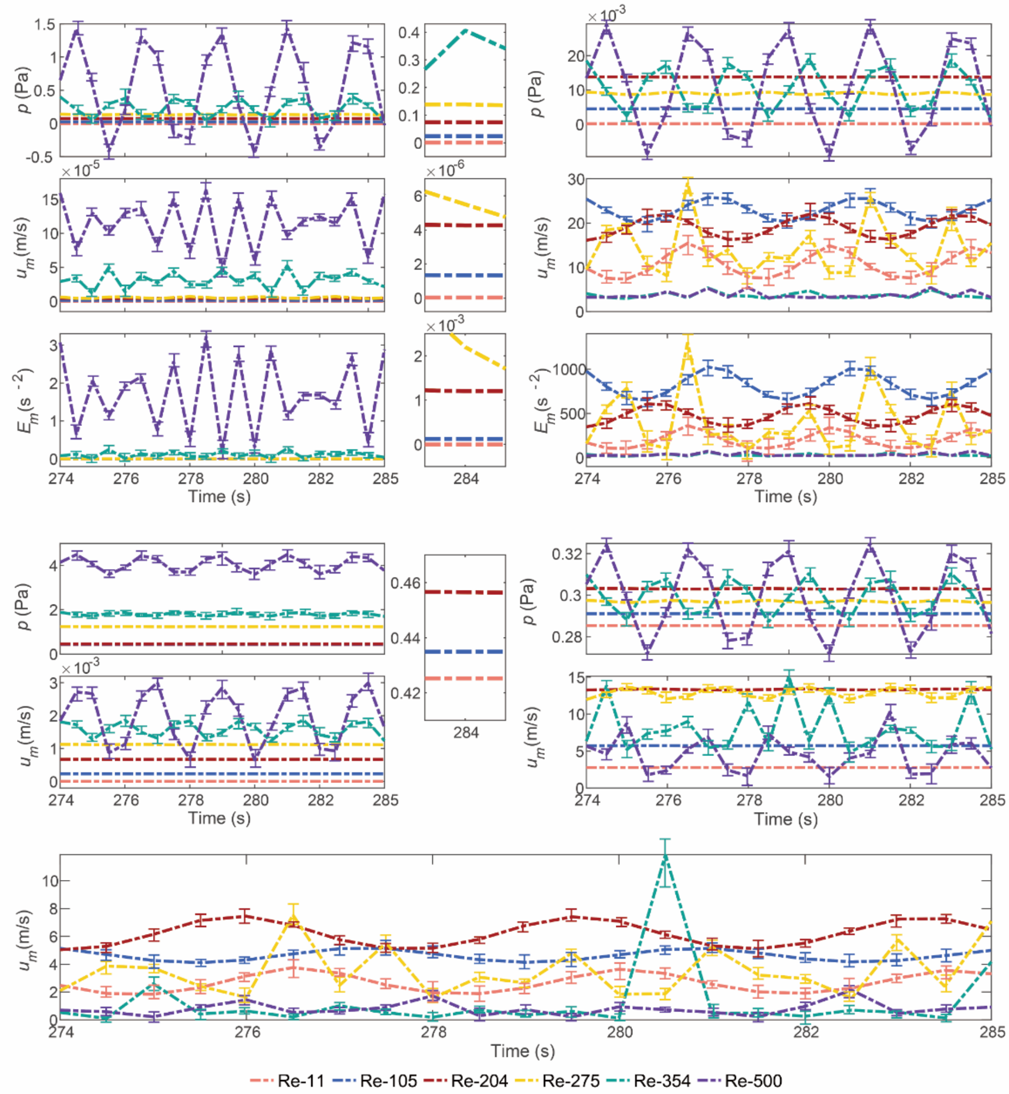

Fig. S8 | Comparison of Internal and Adjacent External Slices for Assessing Asymmetry and Discrepancy in Water Flow within the Skeleton. Fig. S8a illustrates the discrepancies in pressure (**a-1**), velocity magnitude (**a-2**), and vorticity (**a-3**) among the six slices (ABCDEF) within the internal structure. Fig. S8b presents the rate of change in pressure (**b-1**), velocity magnitude (**a-2**), and vorticity (**b-3**) for the internal six slices. Fig. S8c showcases the differences in pressure (**c-1**) and velocity magnitude (**c-2**) between the six internal slices (ABCDEF) and their adjacent external slices (AeBeCeDeEeFe). Fig. S8d displays the rate of change in pressure (**d-1**) and velocity magnitude (**d-2**) for the external six slices. Fig. S8e describes the internal-to-external rate of change in velocity magnitude (**e**). These graphs illustrate the variations in parameters under different Reynolds number conditions when the simulation reaches a steady state. Table S4 provides the specific locations of the internal (ABCDEF) and external (AeBeCeDeEeFe) slices. The parameter differences are calculated using $((A-B)+(C-D)+(E-F)/3$, the rate of change is calculated using $\frac{1}{3}(\frac{A-B}{B}+\frac{C-D}{D}+\frac{E-F}{F})$, and the internal-to-external rate of change is calculated using $\frac{1}{3}(\frac{Ae-Be}{A-B}+\frac{Ce-De}{C-D}+\frac{Ee-Fe}{E-F})$. Error bars indicate standard errors of the mean.

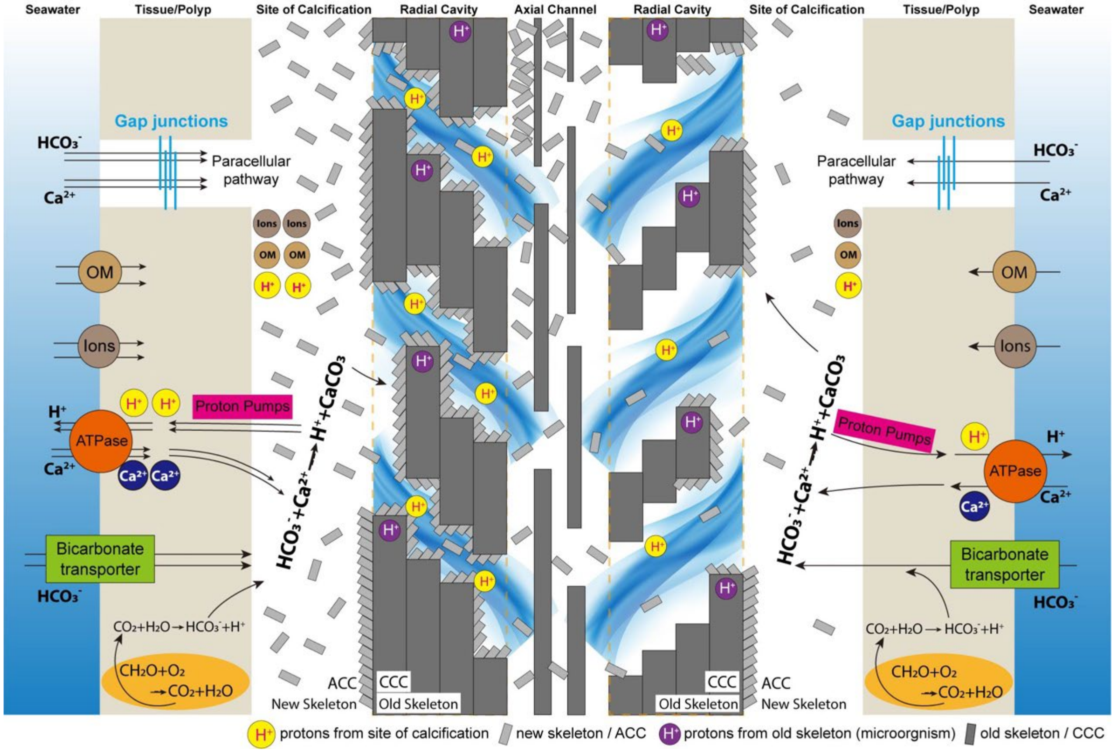

Fig. S9 | Schematic diagram illustrating the impact of internal flow on skeletal growth in Acorpora coral. The asymmetrical flow within the skeleton results from external unidirectional environmental flow upstream. In response, coral tissues exhibit an increased production of amorphous calcium carbonate (ACC)[1] at the calcification site, providing a significant raw material for skeletal growth. In comparison to the downstream side, the asymmetric flow enhances material transport, including organic matrix and protons upstream, fostering dominant skeletal growth on this side. The swift exchange of nutrients and ACC through the calyx at the top facilitates rapid apex growth, sustaining linear extension[2]. In this context, OM represents organic matter[3], and the transport of protons (H^+) and calcium cations (Ca^{2+}) occurs through transcellular and paracellular pathways[4], consuming energy (ATP). ACC denotes nascent calcium carbonate crystals, while CCC signifies the aragonite that has transformed into a dense, old coral skeleton[5]. For a detailed description of coral skeletal precipitation steps, please refer to references[6-8].

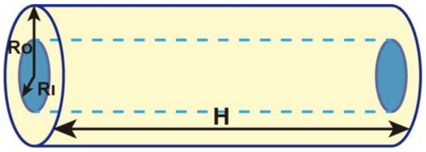

Fig. S10 | Diagram of an equivalent skeleton tube. Diagram of an Equivalent Skeleton Tube: The yellow section illustrates the simplified structure of the skeleton, while the blue section represents pores. The outer diameter signifies the equivalent diameter of the skeleton, the inner diameter represents the equivalent diameter of the pores, and the length corresponds to the equivalent length of the coral branch. The outer diameter is denoted as R_O, the inner diameter as R_I, and the length as H.

Table S1 | Micro-CT setting parameters. The simulation's grid resolution is determined by converting the CT resolution. The original pixels, expressed as coordinates ($\boldsymbol{X_0}, \boldsymbol{Y_0}, \boldsymbol{Z_0}$) in the table, will ultimately be proportionally scaled down to fit the specified simulation domain dimensions ($\boldsymbol{X}, \boldsymbol{Y}, \boldsymbol{Z}$). In this study, the simulation domain aligns with the shrunk pixels (simulation resolution), each represented by LBM cells. The length of each cubic LBM cell, denoted as $\boldsymbol{\delta_x}$, equals the resolution of each simulation $\boldsymbol{\delta_l}$. To optimize computational resources, we opt for $\boldsymbol{\delta_l}$ in simulations, prioritizing efficiency while maintaining accuracy, as opposed to using $\boldsymbol{\delta_{CT}}$.

	Sample.1	*Sample.2*	*Sample.3*
CT settings	100 kV, 10 w, 1.5 s	100 kV, 10 w, 1.5 s	-
Original Pixel $[X_0, Y_0]$	[1985,2026]	[2008,2044]	-
Original tiffs $[Z_0]$	[1998]	[3681]	-
Simulation Domain $[X, Y, Z]$	[499,510,988]	[629,638,739]	[499,510,988]
CT Resolution δ_{CT}	20.465 μm	14.28 μm	-
Simulation Resolution δ_l	81.86 μm	57.12 μm	81.86 μm

Table S2 | Details of ambient flow settings. The preset Reynolds number (calculation details see method) and velocity for the simulations were established according to the natural coral flow environment, taking into account preset viscosity and skeleton diameter. However, the measured Reynolds number, derived from actual simulation results, differs from the preset value due to slight systemic shifts in the simulation conditions. Each model's simulated conditions exhibit these discrepancies. In our analysis, we present a comparative assessment of ambient flow parameter settings for each model, contrasting the preset conditions with the measured ones. The measured maximum velocity corresponds to the velocity at the x=1 slice within the simulation domain, while the measured average velocity represents the average velocity across the entire domain. It is important to note that the actual simulation conditions experienced by the samples deviate slightly from the initially preset conditions, introducing some systematic differences. In defining the Reynolds number for this study, we opted for using the maximum velocity, considering it to be more stable than the average velocity. This choice is motivated by the specific characteristics of the natural coral flow environment under investigation.

	Preset			Measured					Basic settings		
	Re	Physical Velocity (cm/s)	LBM Velocity	Re	LBM Velocity		Physical Velocity (cm/s)		LBM Viscosity	No. of Time Steps	Diameter LBM
					Maximum	Average	Maximum	Average			
S1	9	0.1	0.01	11	0.0125	0.0068	0.0916	0.0498	0.1666	500,000	150
	81	0.9	0.09	105	0.1161	0.0608	0.8510	0.4457			
	153	1.7	0.17	204	0.2267	0.1151	1.6617	0.8437			
	210	2.3	0.14	275	0.1833	0.0943	2.2399	1.1523	0.09996	830,000	
	270	3.0	0.15	354	0.1971	0.1234	2.8895	1.8090	0.0833	1,000,000	
	378	4.2	0.14	500	0.1851	0.0894	4.0703	1.9659	0.0555	830,000	
S1R	9	0.1	0.01	11	0.0125	0.0068	0.0916	0.0498	0.1666	500,000	150
	81	0.9	0.09	105	0.1161	0.0608	0.8510	0.4457			
	153	1.7	0.17	204	0.2267	0.1150	1.6617	0.8430			
	210	2.3	0.14	275	0.1833	0.0947	2.2399	1.1572	0.09996	830,000	
	270	3.0	0.15	354	0.1972	0.1014	2.8910	1.4865	0.0833	1,000,000	
	378	4.2	0.14	500	0.1849	0.0980	4.0660	2.1550	0.0555	830,000	
S1F	81	0.9	0.09	105	0.1161	0.0612	0.8510	0.4486	0.1666	500,000	150
	378	4.2	0.14	500	0.1851	0.0653	4.0703	1.4359	0.0555	830,000	
S1RF	81	0.9	0.09	105	0.1161	0.0612	0.8510	0.4486	0.1666	500,000	150
	378	4.2	0.14	500	0.1849	0.1192	4.0660	2.6212	0.0555	830,000	
S2	19	0.3	0.02	24	0.0251	0.0135	0.2636	0.1418	0.1666	200,000	160
	163	2.4	0.17	218	0.2267	0.1154	2.3804	1.2117			

	327	4.8	0.17	431	0.2246	0.1168	4.7188	2.4540	0.0833	400,000	
S2R	19	0.3	0.02	24	0.0251	0.0135	0.2636	0.1418	0.1666	200,000	160
	163	2.4	0.17	218	0.2267	0.1154	2.3804	1.2117			
	327	4.8	0.17	431	0.2246	0.1175	4.7188	2.4687	0.0833	400,000	
S2F	19	0.3	0.02	24	0.0251	0.0137	0.2636	0.1439	0.1666	200,000	160
	327	4.8	0.17	431	0.2246	0.1192	4.7188	2.5044	0.0833	400,000	
S2RF	19	0.3	0.02	34	0.0251	0.0137	0.2636	0.1439	0.1666	200,000	160
	327	4.8	0.17	431	0.2246	0.1136	4.7188	2.3867	0.0833	400,000	
S3	81	0.9	0.09	105	0.1161	0.0633	0.8510	0.4640	0.1666	100,000	150
	378	4.2	0.14	500	0.1850	0.0883	4.0682	1.9417	0.0555	160,000	

Table S3 | Details of conversion factors. The conversion of parameters for the *Sample.1*, *Sample.2*, and *Sample.3* from Lattice Boltzmann Method (LBM) units to physical units have been conducted using the conversion factors provided above. $\boldsymbol{C_l}$ represents the length conversion factor, obtained by dividing the physical unit length $\boldsymbol{l}$ by the LBM unit length $\boldsymbol{l_L}$. $\boldsymbol{C_\nu}$ is the viscosity conversion factor, derived by dividing the physical viscosity $\boldsymbol{\nu}$ by the LBM viscosity $\boldsymbol{\nu_L}$. $\boldsymbol{C_t}$ stands for the time conversion factor, computed from $\boldsymbol{C_l}$ and $\boldsymbol{C_\nu}$. $\boldsymbol{C_u}$ denotes the velocity conversion factor, calculated from $\boldsymbol{C_l}$ and $\boldsymbol{C_t}$. $\boldsymbol{C_\omega}$ represents the vorticity conversion factor, computed from $\boldsymbol{C_t}$. $\boldsymbol{C_p}$ is the pressure conversion factor, acquired from the density conversion factor and the velocity conversion factor, with the density conversion factor set at 1000 in this paper.

	Re	Length		Viscosity		Time		Velocity		Vorticity		Pressure	
		C_l $[\mu m]$		C_ν $[10^{-6} m^2 \cdot s^{-1}]$		C_t $[\mu s]$		C_u $[m \cdot s^{-1}]$		C_ω $[s^{-1}]$		C_p $[Pa]$	
S1<br>S1R	11	$\frac{l}{l_L}$	81.86	$\frac{\nu}{\nu_L}$	6	$\frac{{C_l}^2}{C_\nu}$	1116.8	$\frac{C_l}{C_t}$	0.0733	$\frac{1}{C_t}$	895.42	$C_\rho {C_u}^2$	5.373
	105												
	204												
	275				10		670.11		0.1222		1492.3		14.93
	354				12		558.42		0.1466		1790.8		21.49
	500				18		372.28		0.2199		2686.2		48.36
S1F<br>S1RF	105				6		1116.8		0.0733		895.42		5.373
	500				18		372.28		0.2199		2686.2		48.36
S2<br>S2R	24		57.12		6		543.78		0.1050		1839.0		11.02
	218												
	431				12		271.89		0.2101		3678.0		44.14
S2F<br>S2RF	24				6		543.78		0.1050		1839.0		11.02
	431				12		271.89		0.2101		3678.0		44.14
S3	11		81.86		6		1116.8		0.0733		895.42		5.373
	500				18		372.28		0.2199		2686.2		46.38

Table S4 | Selected parts at S1 and S1R (coordinates in LBM units) (coordinates in LBM cells). The specific dimensions in LBM units for the selected areas of *Sample.1* and *Sample.1R* are provided here, aiming to facilitate a more detailed analysis of the internal structural characteristics of the coral skeleton. In ParaView, slice sections were selected for asymmetry analysis, encompassing both internal and external asymmetry. Additionally, sections of half-cylinders (e.g., TU/TD) were utilized for further asymmetry analysis, while alternative clip sections (e.g., TT/T/C/B) were employed for the analysis of the axial center channel. Notably, the clip sections of half-cylinders, initially selected as specific parts, were configured as cylinders with particular dimensions taken from various sections (e.g., top/bottom). Subsequently, they were refined by a cuboid (e.g., TU/TD) to attain the desired structure.

<table>
<tr><td rowspan="12">Slice sections
For internal asymmetry</td><td rowspan="6">S1</td><td colspan="2">A = [190-200, 245-255, 90];</td><td rowspan="12">Slice sections
For external asymmetry</td><td rowspan="6">S1</td><td>Ae = [166-176, 245-255, 90];</td></tr>
<tr><td colspan="2">B = [290-300, 245-255, 90];</td><td>Be = [314-324, 245-255, 90];</td></tr>
<tr><td colspan="2">C = [205-215, 245-255, 215];</td><td>Ce = [177-187, 245-255, 215];</td></tr>
<tr><td colspan="2">D = [285-295, 245-255, 215];</td><td>De = [306-316, 245-255, 215];</td></tr>
<tr><td colspan="2">E = [205-215, 245-255, 340];</td><td>Ee = [182-192, 245-255, 340];</td></tr>
<tr><td colspan="2">F = [265-275, 245-255, 340];</td><td>Fe = [297-307, 245-255, 340];</td></tr>
<tr><td rowspan="6">S1R</td><td colspan="2">AR = [300-310, 255-265, 90];</td><td rowspan="6">S1R</td><td>ARe = [300-310, 255-265, 90];</td></tr>
<tr><td colspan="2">BR = [200-210, 255-265, 90];</td><td>BRe = [200-210, 255-265, 90];</td></tr>
<tr><td colspan="2">CR = [285-295, 255-265, 215];</td><td>CRe = [285-295, 255-265, 215];</td></tr>
<tr><td colspan="2">DR = [210-295, 255-265, 215];</td><td>DRe = [210-295, 255-265, 215];</td></tr>
<tr><td colspan="2">ER = [285-295, 250-260, 340];</td><td>ERe = [285-295, 250-260, 340];</td></tr>
<tr><td colspan="2">FR = [220-230, 250-260, 340].</td><td>FRe = [220-230, 250-260, 340].</td></tr>
<tr><td rowspan="5">Clip sections for asymmetry</td><td rowspan="4">S1</td><td rowspan="2">Top</td><td colspan="2" rowspan="2">Cylinder center = [239, 252, 246.5], axis = [0, 0, 1], radius = 40;</td><td colspan="2">Cuboid : TU = [199-219, 211-293, 310-400];</td></tr>
<tr><td colspan="2">Cuboid : TD = [259-279, 211-293, 310-400];</td></tr>
<tr><td rowspan="2">Bottom</td><td colspan="2" rowspan="2">Cylinder center = [245, 254, 246.5], axis = [0, 0, 1], radius = 46.</td><td colspan="2">Cuboid : BU = [199-219, 207-311, 100-300];</td></tr>
<tr><td colspan="2">Cuboid : BD = [271-291, 207-311,100-300].</td></tr>
<tr><td>S1R</td><td>Top</td><td colspan="2">Cylinder center = [261, 259, 246.5], axis = [0, 0, 1], radius = 40;</td><td colspan="2">Cuboid : TUR = [221-241, 218-300, 310-400];</td></tr>
</table>

				Cuboid : TDR = [281-301, 218-300, 310-400];
		Bottom	Cylinder center = [255, 257, 246.5], axis = [0, 0, 1], radius = 46.	Cuboid : BUR = [209-229, 210-304, 100-300];
				Cuboid : BDR = [281-301, 210-304, 100-300].
Clip sections for axial center channel	S1	Top-Tip	Cylinder center = [230, 260, 420], axis = [-0.08, 0.28, 1], radius = 5;	Cuboid : TT = [179-269,137-425, 450-480];
		Top	Cylinder center = [231, 264, 416], axis = [-0.1, 0.12, 1], radius = 5;	Cuboid : T = [168-278, 209-338, 390-450];
		Center	Cylinder center = [240, 258, 331], axis = [-0.09, 0.07, 1], radius = 5;	Cuboid : C = [175-275, 229-309, 250-390];
		Bottom	Cylinder center = [244, 253, 76], axis = [0.01, -0.01, 1], radius = 5.	Cuboid : B = [238-259, 238-259, 1-250].
	S1R	Top-Tip	Cylinder center = [270, 251, 420], axis = [0.08, -0.28, 1], radius = 5;	Cuboid : TTR = [231-321, 91-369, 450-480];
		Top	Cylinder center = [269, 247, 416], axis = [0.1, -0.12, 1], radius = 5;	Cuboid : TR = [222-332, 173-309, 390-450];
		Center	Cylinder center = [260, 253, 331], axis = [0.09, -0.07, 1], radius = 5;	Cuboid : CR = [225-325, 202-282, 250-390];
		Bottom	Cylinder center = [256, 258, 76], axis = [-0.01, 0.01, 1], radius = 5.	Cuboid : BR = [241-262, 252-273, 1-250].

Table S5 | Selected parts for comparison of local defect and center area of *Sample.1*.

S1	Center	Cylinder center = [250, 247, 494], axis = [0, 0, 1], radius = 55;<br>Cuboid = [194-206, 192-203, 250-260];
	Local defect	Sphere center = (210, 205, 382), radius = 8;
S1R	Center	Cylinder center = [250, 264, 494], axis = [0, 0, 1], radius = 55;<br>Cuboid = [194-206, 209-320, 250-260];
	Local defect	Sphere center = (290, 306, 382), radius = 8;

Table S6 | Selected parts for Fig. 1D for *Sample.1*

S1	Cylinder center = [245, 255, 494], axis = [0, 0, 1], radius = 42;<br>Cuboid = [202-288, 240-255, 175-275];

Table S7 | Selected parts for Fig. 4a-c.

S1	Cuboid = [160-310, 170-320, 380-390]
S1F	
S3	Cuboid = [140-360, 150-370, 380-390]

Table S8 | Selected parts for Sample. 1R of vorticities, corresponding to Table S7.

S1R	Cuboid = [190-340, 201-331, 380-390]
S1FR	
S3	Cuboid = [140-360, 150-370, 380-390]

Table S9 | Selected parts for *Sample. 1R* of vorticities, corresponding to Table S8. The central undamaged site was selected due to its relatively stable and consistent structure, known from the top-tip part, making it a representative segment of the entire skeleton. The calculation of flow flux in this area is based on the residence time in the central undamaged parts, constituting approximately 95% of the total flux, while the flux within the local defects is fully utilized at 100%. Notably, discernible vortices and turbulence are observed at Re = 354 for *Sample.1* and Re = 500 for *Sample.1R* (in Movie S2). At the same location, the flow rate gradually increases with the rise in Reynolds number, particularly when turbulence occurs, resulting in a more pronounced augmentation of the flow rate. This suggests that the occurrence of turbulence significantly influences fluid motion, further propelling an increase in the flow rate. To enhance result accuracy, the average outcomes from the period between 278 and 280 seconds were employed. In comparison to the typical structure in central undamaged sites, the internal flow flux within local defects lacking coral-effective tissue covers is higher.

In this study, residence time (or flushing time) is computed through streamlines entering the inner pores of the coral, excluding those generated by inner dead pores (approximately 5% of the total streamlines). We conducted a comparison of the residence time for groups encompassing 80%, 90%, 95%, 98%, and 100% of all streamlines. It is important to note that the residence time for groups exceeding 95% is unstable, given that the velocity near the end of dead pores approaches 0, while groups with ≤95% of streamlines display relatively stable characteristics.

Re	*Sample.1* – Q (mm^3/h)				*Sample.1R* - Q (mm^3/h)			
	CU	%	LD	%	CU – R	%	LD – R	%
11	2.47875	95.36	42.3640	100	30.1543	95.67	18.7288	100
105	34.9006	95.70	117.680		50.5239	95.47	131.239	
204	158.060	95.53	1918.84		154.523	95.28	271.059	
275	219.563	95.56	2445.43		168.459	95.02	344.725	
354	1524.91	95.38	13871.3		198.155	95.54	603.837	
500	4305.88	95.42	43062.1		2922.57	95.54	16361.3	

Table S10 | Residence time (flushing time) analysis: *Sample.1* within the entire coral skeleton and a typical calyx. With the increase in Reynolds number, we conducted a comparative analysis, focusing on the residence time (flushing time) in two cases: the 95% residence time within all pores of the entire coral skeleton and the 95% residence time within a typical calyx pore. For the internal pores of the entire skeleton, we processed images of S1 and S1F, releasing 100,000 particle traces within a cylindrical volume covering the entire skeletal structure. As for the typical calyx pore, using Fig. 4d as a reference, we released 1,000 particle traces within a spherical volume (centered at 231, 291, 399) with a radius of 8 (in LBM units). The residence time of water flow was calculated based on particle traces, and the specific calculation method is detailed in the method. $\boldsymbol{R}_{\mathbf{E}}$ is the residence time of the entire coral skeleton, $\boldsymbol{R}_{\mathbf{T}}$ is the residence time of the typical calyx, and $\frac{\boldsymbol{R}_{\mathbf{E}}}{\boldsymbol{R}_{\mathbf{T}}}$ is the ratio of them.

	Entire coral skeleton		Typical calyx		$\frac{R_E}{R_T}$
Re	Residece time ($\times 10^5$ s)	%	Residece time ($\times 10^3$ s)	%	
11	1.0149	95.11	8.8057	95.42	11.5
105	0.0506	95.29	0.3290	95.51	15.4
204	0.0194	95.53	0.1002	95.51	19.4
275	0.0079	95.55	0.0779	95.12	10.1
354	0.0011	95.15	0.0266	95.60	4.1
500	0.0004	95.37	0.0040	95.53	10.0

Table S11 | Dissolution of coral skeleton aragonite of *Sample.1* in 2003 and 2100. The parameters $\boldsymbol{R_O}$, $\boldsymbol{R_I}$, and H are the size parameters of the equivalent calcium carbonate tube, corresponding to the outer diameter, inner diameter, and length, respectively (see Fig. S10). The parameter $\boldsymbol{n_0}$ stands for the initial porosity. The symbol $\boldsymbol{pH_{surface\ seawater}}$ represents the predicted pH of surface seawater[9], while $\boldsymbol{pH_{proton\ source}}$ indicates the predicted pH in the proton source place[10] (from a low pH condition). $\boldsymbol{\Omega_{Aragonite} = \frac{[Ca^{2+}][CO_3{}^{2-}]}{K_{sp}{}^{*}}}$ is the aragonite saturation in seawater[11]. Aragonite will dissolve spontaneously when $\boldsymbol{\Omega < 1}$, and the value of $\boldsymbol{\Omega_{Aragonite}}$ here considering the low pH in the sediment is undersaturated. $\boldsymbol{Q_{\mathrm{whole}}}$ represents the flux flow through the inner pores of the entire coral skeleton, calculated by residence time of the streamlines. The variables r, N, and G donate the aragonite dissolution rate, the skeleton's porosity change ratio, and the structure's rigidity change ratio, respectively. Additionally, oe and ie represent scenario 1 and scenario 2 (see methods), respectively. We conducted a detailed comparative study, systematically analyzing the changes in dissolution rate $\boldsymbol{r_{2100}/r_{2023}}$, porosity $\boldsymbol{N_{2100}/N_{2023}}$, and stiffness $\boldsymbol{G_{2100}/G_{2023}}$ of coral porous aragonite skeletons between the years 2023 and 2100 under two acidification assumptions. This analysis aims to demonstrate the potential impacts of future ocean acidification on essential components of coral ecosystems.

	Sample.1	*Sample.2*			2023	2100
R_O (mm)	4.66	3.94		$pH_{surface\ seawater}$ [9]	8.07	7.87
R_I (mm)	2.82	2.54		$pH_{proton\ source}$ [10]	7.52	7.37
H (mm)	38.3	24.1		$\Omega_{Aragonite}$ [12]	0.87	0.44
n_0	0.37	0.42				

	Re	Q_{whole} ($m^3\ s^{-1}$)	%	2023						2100					
				oe			ie			oe			ie		
				r ($mol\ m^{-3}\ s^{-1}$)	N (%)	G (%)	r ($mol\ m^{-3}\ s^{-1}$)	N (%)	G (%)	r ($mol\ m^{-3}\ s^{-1}$)	N (%)	G (%)	r ($mol\ m^{-3}\ s^{-1}$)	N (%)	G (%)
S1	11	9.76×10^{-12}	95.11	3.08×10^{-10}	3.60×10^{-5}	2.83×10^{-5}	5.53×10^{-6}	0.647	0.507	4.35×10^{-10}	5.09×10^{-5}	3.99×10^{-5}	3.55×10^{-4}	45.76	34.10
	105	1.96×10^{-10}	95.29	6.19×10^{-9}	7.23×10^{-4}	5.67×10^{-4}				8.74×10^{-9}	1.02×10^{-3}	8.01×10^{-4}			
	204	5.11×10^{-10}	95.53	1.61×10^{-8}	1.89×10^{-3}	1.48×10^{-3}				2.28×10^{-8}	2.66×10^{-3}	2.09×10^{-3}			
	275	1.26×10^{-9}	95.55	3.97×10^{-8}	4.63×10^{-3}	3.64×10^{-3}				5.60×10^{-8}	6.54×10^{-3}	5.13×10^{-3}			
	354	9.41×10^{-9}	95.15	2.97×10^{-7}	3.47×10^{-2}	2.72×10^{-2}				4.20×10^{-7}	4.90×10^{-2}	3.85×10^{-2}			
	500	2.41×10^{-8}	95.37	7.60×10^{-7}	8.88×10^{-2}	6.97×10^{-2}				1.07×10^{-6}	1.25×10^{-1}	9.84×10^{-2}			
S2	24	1.33×10^{-10}	95.42	8.22×10^{-9}	9.60×10^{-4}	9.27×10^{-4}	6.14×10^{-6}	0.718	0.693	1.16×10^{-8}	1.36×10^{-3}	1.31×10^{-3}	3.93×10^{-4}	51.17	45.90
	218	3.81×10^{-10}	95.63	2.36×10^{-8}	2.75×10^{-3}	2.66×10^{-3}				3.33×10^{-8}	3.89×10^{-3}	3.76×10^{-3}			
	431	1.75×10^{-9}	95.60	1.08×10^{-7}	1.26×10^{-2}	1.22×10^{-2}				1.53×10^{-7}	1.78×10^{-2}	1.72×10^{-2}			

	r_{2100}/r_{2023}		N_{2100}/N_{2023}		G_{2100}/G_{2023}	
	Scenario 1, oe	Scenario 2, ie	Scenario 1, oe	Scenario 2, ie	Scenario 1, oe	Scenario 2, ie
S1	1.41	64.20	1.41	70.74	1.41	67.22
S2	1.41	64.01	1.41	71.22	1.41	66.22

Supplementary References

1 Mass, T. *et al.* Amorphous calcium carbonate particles form coral skeletons. *Proceedings of the National Academy of Sciences* **114**, E7670-E7678 (2017).

2 Mollica, N. R. *et al.* Ocean acidification affects coral growth by reducing skeletal density. *Proceedings of the National Academy of Sciences* **115**, 1754-1759 (2018).

3 Von Euw, S. *et al.* Biological control of aragonite formation in stony corals. *Science* **356**, 933-938 (2017).

4 Clark, M. S. Molecular mechanisms of biomineralization in marine invertebrates. *Journal of Experimental Biology* **223**, jeb206961 (2020).

5 Guo, W. Seawater temperature and buffering capacity modulate coral calcifying pH. *Scientific Reports* **9**, 1189 (2019).

6 Jokiel, P. L. The reef coral two compartment proton flux model: A new approach relating tissue-level physiological processes to gross corallum morphology. *Journal of Experimental Marine Biology and Ecology* **409**, 1-12 (2011).

7 Comeau, S. *et al.* Coral calcifying fluid pH is modulated by seawater carbonate chemistry not solely seawater pH. *Proceedings of the Royal Society B: Biological Sciences* **284**, 20161669 (2017).

8 Tambutté, S. *et al.* Coral biomineralization: from the gene to the environment. *Journal of Experimental Marine Biology and Ecology* **408**, 58-78 (2011).

9 Brewer, P. G. Ocean chemistry of the fossil fuel CO2 signal: The haline signal of "business as usual". *Geophysical Research Letters* **24**, 1367-1369 (1997).

10 Andersson, A. J. & Gledhill, D. Ocean acidification and coral reefs: effects on breakdown, dissolution, and net ecosystem calcification. *Annual review of marine science* **5**, 321-348 (2013).

11 Feely, R. A., Sabine, C. L., Hernandez-Ayon, J. M., Ianson, D. & Hales, B. Evidence for upwelling of corrosive" acidified" water onto the continental shelf. *science* **320**, 1490-1492 (2008).

12 Morse, J. W., de Kanel, J. & Harris, K. Dissolution kinetics of calcium carbonate in seawater; VII, The dissolution kinetics of synthetic aragonite and pteropod tests. *American Journal of Science* **279**, 488-502 (1979).
